# Origami Metamaterials Based on Low‐Melting‐Point Alloy Phase Transition: Breaking the Trade‐Off Between Reusability and Energy Absorption Quality

**DOI:** 10.1002/advs.76536

**Published:** 2026-07-09

**Authors:** Yupeng Liu, Wei Zhao, Chengjun Zeng, Jiuming Fan, Yanju Liu, Jinsong Leng

**Affiliations:** ^1^ Department of Astronautical Science and Mechanics Harbin Institute of Technology (HIT) Harbin P. R. China; ^2^ Guangzhou Institute of Future Additive Manufacturing Guangzhou P. R. China; ^3^ Suzhou Research Institute Harbin Institute of Technology (HIT) Suzhou P. R. China; ^4^ Center for Composite Materials and Structures Harbin Institute of Technology (HIT) Harbin P. R. China

**Keywords:** energy absorption, liquid metal, origami metamaterials, shape memory, tunability

## Abstract

Mechanical metamaterials provide a promising platform for designing energy‐absorbing materials. However, the trade‐off between reusability and energy absorption quality limits the overall performance of existing energy‐absorbing metamaterials. To address this challenge, an origami metamaterial based on the low‐melting‐point alloy phase transition is proposed in this study, constructed by integrating a low‐melting‐point alloy skeleton into an elastomeric shell. In terms of performance, the metamaterial achieves an energy absorption capacity of 41.5 kJ·m^−^^2^, a crushing force stability of 0.838, and a reusability ratio of 97.8%. This outstanding overall performance stems from a multilevel synergistic design. At the material level, plastic deformation of the metal skeleton provides high energy absorption. Meanwhile, the heat‐induced solid–liquid phase transition of the low‐melting‐point alloy, together with the hyperelasticity of the elastomeric shell, enables high structural recoverability. At the unit‐cell level, tailoring the geometric parameters yields a stable force response, and the diamond origami configuration further enhances energy absorption quality. At the multi‐cell system level, eliminating deformation coupling between layers significantly enhances the deformation mode stability of multilayer metamaterials, thereby extending the effective compression stroke. Overall, the metamaterial simultaneously achieves high‐quality energy absorption and high reusability, showing great potential for engineering applications that require repeated energy absorption.

## Graphical abstract

This work presents an origami metamaterial that integrates a low‐melting‐point alloy skeleton into an elastomeric shell. It dissipates energy through the metal's plastic deformation and recovers through the solid–liquid phase transition of the alloy together with the hyperelasticity of the shell. This design breaks the long‐standing trade‐off between reusability and energy absorption quality that exists in conventional energy‐absorbing materials.

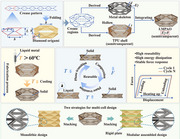

## 1 Introduction

Mechanical metamaterials exhibit counterintuitive properties that are not found in conventional materials [1], such as ultralight weight [2, 3], negative Poisson's ratio [4, 5, 6], programmability [7, 8, 9], and reconfigurability [10, 11, 12]. These properties arise from rationally designed microstructures and carefully selected constituent materials. In recent years, research on mechanical metamaterials has gradually expanded from the tuning of individual mechanical properties to multifunctional synergistic design [13, 14, 15]. In addition, mechanical metamaterials offer a promising platform for the design of energy‐absorbing materials [14, 16, 17, 18, 19, 20, 21, 22].

Energy‐absorbing materials can effectively protect individuals and objects from unpredictable impacts. Their fundamental function is to absorb or dissipate the incoming impact energy, thereby limiting the peak force transmitted to protected targets below a safe threshold [23]. Simultaneously enhancing the energy absorption capacity and the stability of the force response is the key design objective for energy‐absorbing metamaterials [24]. These two indicators are integrated into “energy absorption quality” to provide a comprehensive evaluation of the single‐impact energy absorption performance in this work. Considerable research has been devoted to enhancing energy absorption quality. For example, diamond origami metamaterials employ prefolded patterns to induce compressive deformation into a diamond mode, which doubles the number of mobile plastic hinges and thereby enhances the energy absorption capacity. Meanwhile, the prefolded pattern itself can be regarded as a macroscopic geometric defect and contributes to reducing the initial peak crushing force (IPCF) and improving the stability of the force response [25]. Similarly, performance‐oriented and deformation‐constrained dual‐topology metamaterials integrate the advantages of stretch‐dominated structures and bending‐dominated structures, achieving both high stress levels and stress stability [26]. The metamaterials mentioned above are typically made of metals, and their excellent performance relies on the energy dissipation mechanisms of the constituent materials. These mechanisms include fracture and plastic deformation, enabling the materials to dissipate substantial energy through dislocation motion or bond breakage at the atomic level. However, due to the irreversibility of these energy dissipation mechanisms, such metamaterials are typically limited to one‐time usage.

**FIGURE 1:**
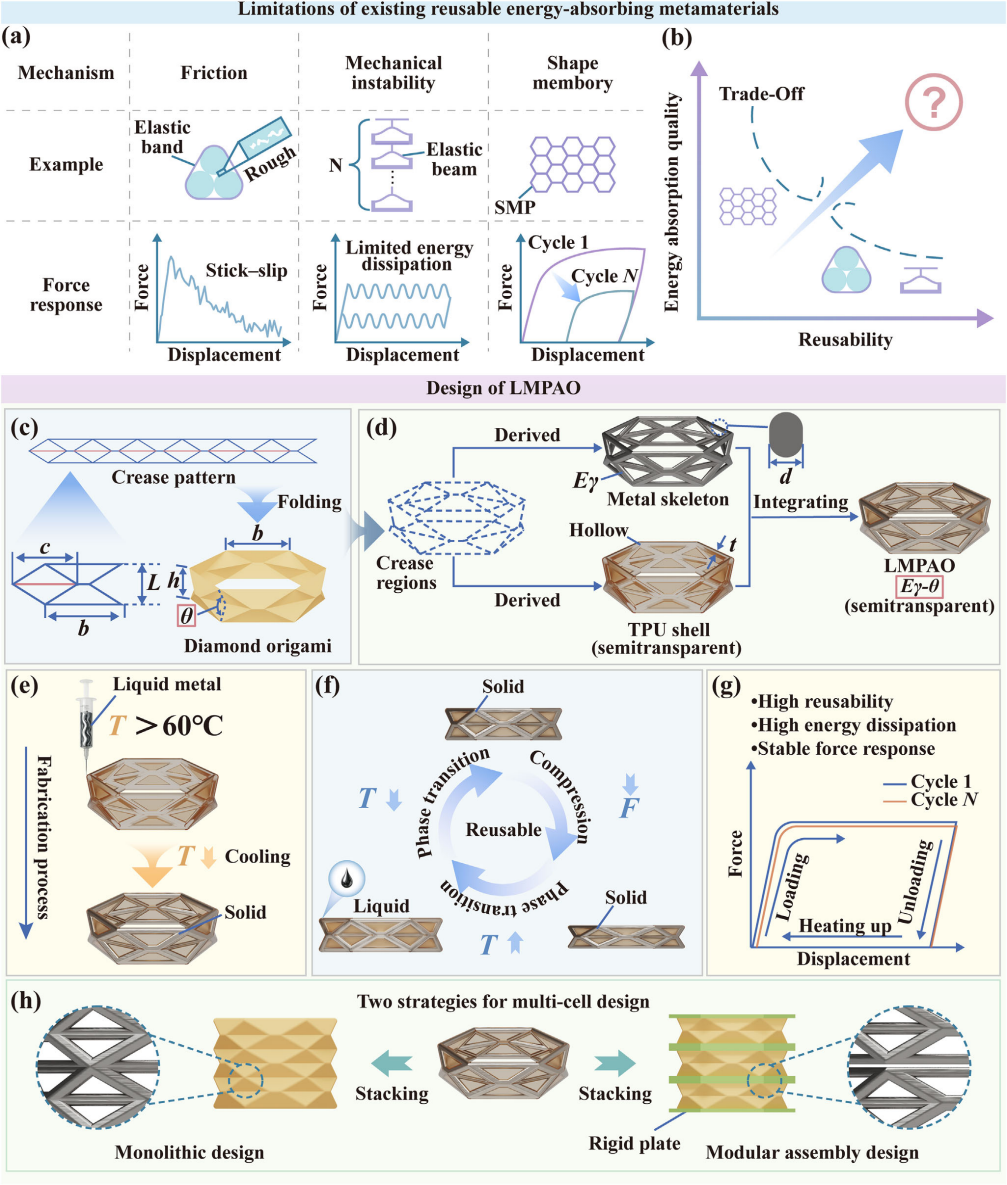
Limitations of existing reusable energy‐absorbing metamaterials and the design of LMPAO. (a) Three typical reusable energy absorption mechanisms and the corresponding structural force responses. (b) The trade‐off between reusability and energy absorption quality in existing reusable energy‐absorbing metamaterials. (c) Schematic of the construction process of the diamond origami structure. (d) Schematic of the construction process of the LMPAO unit cell. The diamond origami TPU shell and the metal skeleton are geometrically complementary. The semitransparent rendering clearly reveals the shell structure. (e) Fabrication process of the LMPAO unit cell. (f) Reusable energy absorption mechanism of the LMPAO. (g) Force response characteristics of the optimized LMPAO. (h) Two multi‐cell design strategies for LMPAO. The shells of the multi‐cell structures are rendered opaque to match their actual visible appearance.

To overcome the limitation of one‐time usage, researchers have developed a series of reusable energy‐absorbing metamaterials. As shown in Figure 1a, existing reusable energy‐absorbing metamaterials mainly rely on three types of reusable mechanisms: the friction mechanism [23, 27, 28, 29, 30], the bistable or mechanical instability mechanism [31, 32, 33, 34, 35, 36, 37, 38, 39, 40, 41, 42, 43, 44], and the shape memory mechanism [45, 46, 47, 48, 49, 50, 51, 52, 53]. Generally, friction‐based metamaterials convert part of the external impact energy into elastic strain energy stored in the elastomer, while dissipating the other part as heat through friction. However, the stick‐slip friction phenomenon may cause severe oscillations in the force–displacement curve of such metamaterials [54], and the crushing force stability is usually low. Bistable metamaterials exhibit two stable states with distinct energy levels, and the transition between these states is accompanied by energy absorption or release. Metamaterials based on mechanical instability exhibit hysteresis loops by axially stacking a large number of buckling units, thereby enabling self‐recoverable energy dissipation. However, the constituent materials of such structures are generally elastomers with low stiffness, resulting in limited energy dissipation. Metamaterials based on shape memory alloys (SMA) and shape memory polymers (SMP) dissipate energy through hysteretic behavior associated with phase transformation or viscoelasticity, exhibit high energy dissipation capability, and can recover their deformed shape through the shape memory effect. However, they often exhibit mechanical performance degradation after cyclic loading, thus showing limited reusability. In summary, existing reusable energy‐absorbing metamaterials either struggle to achieve high energy absorption quality (metamaterials based on friction or mechanical instability) or fail to maintain good reusability (metamaterials based on SMA and SMP), and they fall within the trade‐off region shown in Figure 1b.

To break this trade‐off, we propose an origami metamaterial based on the low‐melting‐point alloy (LMPA) phase transition mechanism. In light of the fact that strain concentrates at the creases during compression of the metallic diamond origami structure [24], we inject molten LMPA into the hollow crease regions of the diamond origami TPU shell. After the injected liquid metal cools and solidifies, a low‐melting‐point alloy (LMPA) embedded origami metamaterial is formed, referred to as LMPAO. The LMPA–TPU composite system provides a mechanism that enables the structure to simultaneously achieve high energy absorption and high reusability. At room temperature, the metal skeleton embedded in the TPU shell remains in the solid state and absorbs a large amount of energy through plastic deformation during compression. Upon heating, the metal skeleton undergoes a solid–liquid phase transition, and the hyperelastic TPU shell provides sufficient restoring force to enable the recovery of the LMPAO metamaterial. At the structural level, the diamond origami pattern induces the LMPAO to follow the diamond deformation mode, thereby further improving its reusability and energy absorption quality. In addition, we systematically investigate the tuning effect of geometric parameters on the mechanical response of LMPAO, yielding unit cells with a stable force response and an adjustable load plateau. To address the deformation mode degradation that easily occurs in multilayer LMPAO, we propose optimization strategies that enhance the stability of the deformation mode, thereby extending the compression stroke and increasing the energy absorption per unit projected area. Here, the proposed LMPAO achieves an energy absorption capacity of up to 41.5 kJ·m^−^^2^ and a crushing force stability of 0.838 while maintaining a reusability ratio of 97.8%, breaking the trade‐off between high reusability and high energy absorption quality in conventional reusable energy‐absorbing metamaterials.

## 2 Results

### 2.1 Design of the Low‐Melting‐Point Alloy Embedded Origami Metamaterial (LMPAO)

Previous studies have shown that, during compression, the deformation of diamond origami structures can follow a stable and predictable diamond deformation mode, and the strain is mainly concentrated in the crease regions [25]. This deformation feature matches the design requirements of the LMPAO. On the one hand, the stable diamond mode helps the structure maintain a reproducible deformation path during cyclic loading, which is essential for achieving a high degree of overlap among the force–displacement curves from different cycles. On the other hand, strain concentration at the creases provides well‐defined locations for placing the LMPA skeleton, allowing the high‐energy‐absorbing material to coincide with the main deformation regions. In addition, the regular top and bottom interfaces of the diamond origami unit cell facilitate subsequent modular stacking. Therefore, we adopt diamond origami as the base configuration for the proposed LMPAO. As illustrated in Figure 1c, folding the crease pattern along the predefined creases and connecting the two end boundaries yields a closed three‐dimensional diamond origami structure. In the flat crease pattern, blue lines and red lines represent mountain and valley creases, respectively. The enlarged region represents a single unit of the pattern, consisting of a pair of vertically symmetric rhombuses and a pair of vertically symmetric isosceles trapezoids. The crease pattern contains six basic units, and its geometric parameters are uniquely determined by the valley crease length *c*, the bottom base length *b* of the trapezoid, and the vertical height *L* of the crease pattern. The diamond origami structure has zero degrees of freedom, and its geometric parameters are uniquely determined by three independent variables: the layer height *h*, the dihedral angle *θ*, and the base length *b* (see the detailed derivation in Section S1).

Figure 1d illustrates the construction process of the LMPAO unit cell. Considering that stresses concentrate at the creases during compression of metallic diamond origami structures [24], we construct a metal skeleton in the crease regions and a TPU shell with predefined hollow channels at the creases. By integrating these two components, we obtain a complete LMPAO unit cell. The TPU shell thickness *t*, the metal skeleton thickness *d*, and its width level *Eγ*, together with the three independent variables that define the diamond origami configuration, constitute six independent variables that uniquely determine the geometry of the LMPAO. In this work, since *b* and *h* are fixed and *d* is functionally dependent on *t* (*d* = *t* − 2), the number of independent variables is reduced to three: *Eγ*, *θ*, and *t*. Unless otherwise specified, *t* is set to 5 mm, and we denote the structure as *Eγ*‐*θ*. The modeling method of the LMPAO and the definition of the width levels are detailed in Section S2, and the schematic diagrams of the structures corresponding to the naming convention are provided in Figure S3.

Figure 1e illustrates the fabrication process of the LMPAO. First, we fabricate the TPU shell via 3D printing. Then, we heat the LMPA to 60°C to liquefy it and use a syringe to inject the liquid metal into the TPU shell. Finally, the composite structure is allowed to cool naturally. As the injected liquid metal solidifies, the fabrication of the LMPAO metamaterial is completed, with the metal skeleton cleverly embedded within the TPU shell.

Figure 1f illustrates the reusable energy absorption mechanism of the LMPAO metamaterial. At room temperature, the embedded metal skeleton remains in the solid state. Its metallic nature enables the structure to absorb substantial energy through plastic deformation under compression, while also suppressing structural rebound to some extent. Upon heating to 60°C, the metal skeleton undergoes a solid–liquid phase transition, and its plastically deformed state is eliminated. At this point, the hyperelastic TPU shell provides sufficient driving force for structural recovery. Finally, the structure is left to cool naturally, and once the liquid metal fully solidifies, the LMPAO metamaterial fully recovers. Figure 1g illustrates the force response characteristics of the optimized LMPAO. After multiple cyclic loadings, its mechanical performance exhibits almost no degradation, demonstrating high reusability. Within a single loading–unloading cycle, the LMPAO achieves high energy absorption while maintaining a stable force response, reflecting high energy absorption quality.

Finally, to extend the compression stroke, two multi‐cell design strategies for LMPAO are proposed. As shown in Figure 1h, the first strategy is to axially stack multiple unit cells to form a monolithic multilayer structure (left). The second strategy is to use rigid plates with grooves to connect and constrain multiple unit cells, thereby constructing a modular structure (right). These two multi‐cell designs further give rise to three strategies for enhancing deformation mode stability, which are discussed in detail in Section 2.4.

### 2.2 Tunable Mechanical Response of the LMPAO Unit Cell

**FIGURE 2:**
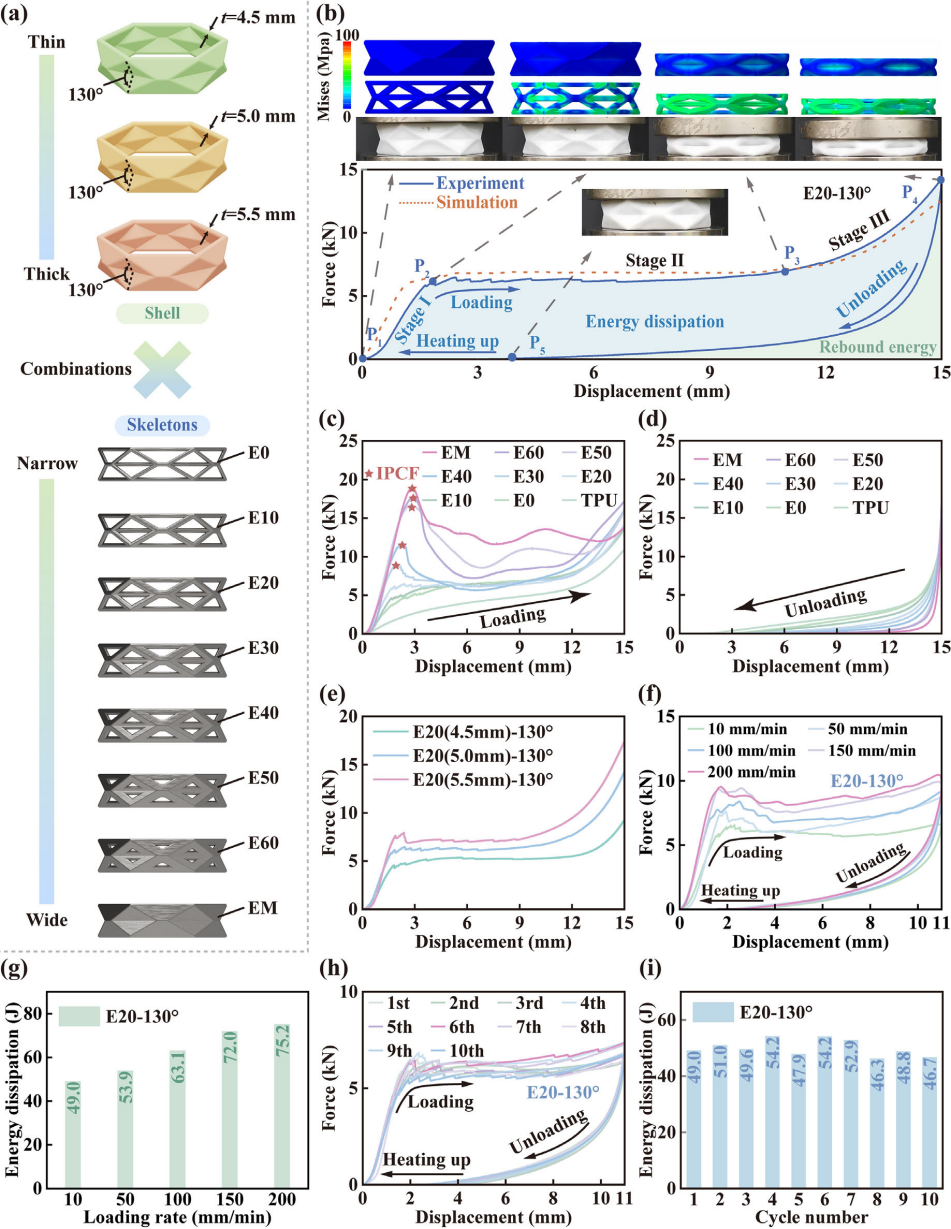
Tunable mechanical response of the LMPAO unit cell and its energy dissipation characteristics. (a) Schematic of the LMPAO unit‐cell composition and its tunable parameters. These parameters include the shell thickness and the width level of the metal skeleton. (b) Force–displacement curve from the quasi‐static compression–unloading test of E20‐130°, experimental snapshots of the structure at key stages, and stress distributions of the TPU shell and the metal skeleton. The blue‐shaded area indicates the energy dissipation, while the green‐shaded area represents the rebound energy, which may cause secondary damage and should be minimized. (c) Force–displacement curves obtained during the first quasi‐static compression of the LMPAO embedded with metal skeletons of different width levels. The dihedral angle is fixed at 130° and is therefore omitted from the legend. (d) Quasi‐static unloading curves corresponding to the compression curves. (e) Compression curves of LMPAO structures with different thicknesses, all embedded with metal skeletons of width level E20. (f) Force–displacement curves of E20‐130° at different loading rates. (g) Energy dissipation in a single cycle of E20‐130° at different loading rates. (h) Force–displacement curves of ten cyclic tests within the effective stroke of E20‐130°. (i) Energy dissipation in each of the ten cyclic tests.

To meet diverse energy absorption requirements, we systematically investigate the tunable mechanical behavior of the LMPAO unit cell. First, we construct a series of LMPAO unit cells with different geometric parameters. As shown in Figure 2a, at a fixed origami dihedral angle of 130°, we specify three shell thicknesses: 4.5, 5.0, and 5.5 mm. For each thickness, eight metal skeletons with different width levels are designed, and an additional TPU origami structure without a metal skeleton is included as a control. A total of 27 structures are obtained. Subsequently, each structure undergoes three cyclic compression–unloading–recovery tests to evaluate the variation of the mechanical response with structural parameters. Energy absorption evaluation indicators are provided in Section S4, and the raw experimental data are available in Figures S5–S7.

To elucidate the energy dissipation mechanism of the LMPAO metamaterial, as illustrated in Figure 2b, we present the mechanical response of the E20‐130° specimen (5 mm thick) during quasi‐static cyclic tests. The force–displacement curve obtained from the quasi‐static compression process can be divided into three stages, beginning with a linear stage (P1‐P2). The force increases approximately linearly as compression proceeds. This stage is extremely short, involves only minor deformation, and contributes little to energy absorption. The linear stage ends at point P2, where the force–displacement curve enters the second stage: the plateau stage (P2‐P3). The stable reaction force and long compression stroke make it the main stage of energy absorption. At point P3, the structural stress concentrates in the rhombic region of the metal skeleton, while the stress in the TPU shell is negligible by comparison, indicating that the metal skeleton plays the primary role in energy absorption. After point P3, the force–displacement curve enters the densification stage (P3‐P4). As the LMPAO is gradually densified, the reaction force rises sharply and is likely to exceed the tolerance limit of the protected object, indicating that the protective function has failed at this stage. Throughout the compression process, the pre‐folded pattern consistently guides the deformation of the LMPAO, causing the rhombic regions to collapse inward and the trapezoidal regions to expand outward. We define this deformation mode as the diamond mode.

Point P4 corresponds to the maximum displacement set in the quasi‐static compression test. Beyond this point, the quasi‐static unloading stage (P4‐P5) begins, during which the LMPAO rebounds and releases part of the absorbed energy. After unloading, we place the LMPAO in a 60°C oven and heat it for 10 min, during which the metal skeleton melts and the structure recovers its shape. We then allow it to cool at room temperature. Once the liquefied metal has fully solidified, the LMPAO is fully recovered, completing a closed compression–unloading–recovery cycle (see Movie S1).

Figure 2c presents the force–displacement curves of the LMPAO (5 mm thick) embedded with metal skeletons of different width levels during the first quasi‐static compression. For width levels of the metal skeletons less than or equal to E20, the force–displacement curves do not exhibit an initial peak crushing force (IPCF), and the crushing force stability increases as the width of the metal skeletons increases. Once the width level of the metal skeletons exceeds E20, the force–displacement curves exhibit an IPCF. In this case, increasing the width of the metal skeletons enhances the structural stiffness and thus raises the buckling threshold, resulting in a higher IPCF but reduced crushing force stability. In summary, adjusting the width of the metal skeletons can alter the structural stiffness, thereby controlling the buckling threshold, the IPCF, and the crushing force stability. At the width level of E20 for the metal skeleton, the corresponding force–displacement curve exhibits no IPCF and achieves the highest crushing force stability, representing an ideal energy absorption profile.

Figure 2d shows the force–displacement curves during the corresponding quasi‐static unloading process. Due to the hyperelasticity of the TPU shell and the elastoplastic behavior of the metal skeleton, both components rebound in the initial stage of unloading. This rebound behavior leads to a steep slope on the curve at this stage. However, in the later stage of unloading, the TPU shell continues to rebound, while the elastic deformation of the metal skeleton has already recovered. The rebound of the TPU shell is hindered by the metal skeleton, leading to a relatively low slope of the unloading curve during this stage. As the overall structural rebound is primarily driven by the TPU shell, a lower metal content results in greater energy release during rebound.

Figure 2e presents the compression curves of LMPAO structures with different thicknesses, all embedded with metal skeletons of width level E20. Each LMPAO structure under this condition exhibits excellent crushing force stability, and the height of the force plateau increases with increasing structural thickness. This enables the tailored selection of energy‐absorbing structures based on the protection requirements of specific targets.

Figure 2f shows the force–displacement curves of E20‐130° at different loading rates. As the loading rate increases, the area enclosed by the loading–unloading curves of the LMPAO increases, while the loading curves still maintain a relatively stable force response. As shown in Figure 2g, the energy dissipation of the LMPAO increases with the loading rate. Therefore, the structure may exhibit enhanced energy absorption performance under impact conditions.

To investigate whether the structure remains reusable after multiple cycles, we selected the E20‐130° (5 mm thick), which exhibits the highest crushing force stability, and subjected it to ten cyclic tests within the compression stroke. As shown in Figure 2h, the ten cyclic curves exhibit a high degree of overlap. Furthermore, Figure 2i presents the energy dissipation in each cycle. In the tenth cycle, the energy dissipation decreases to only 95.3% of that in the first cycle, demonstrating the excellent reusability of the LMPAO.

**FIGURE 3:**
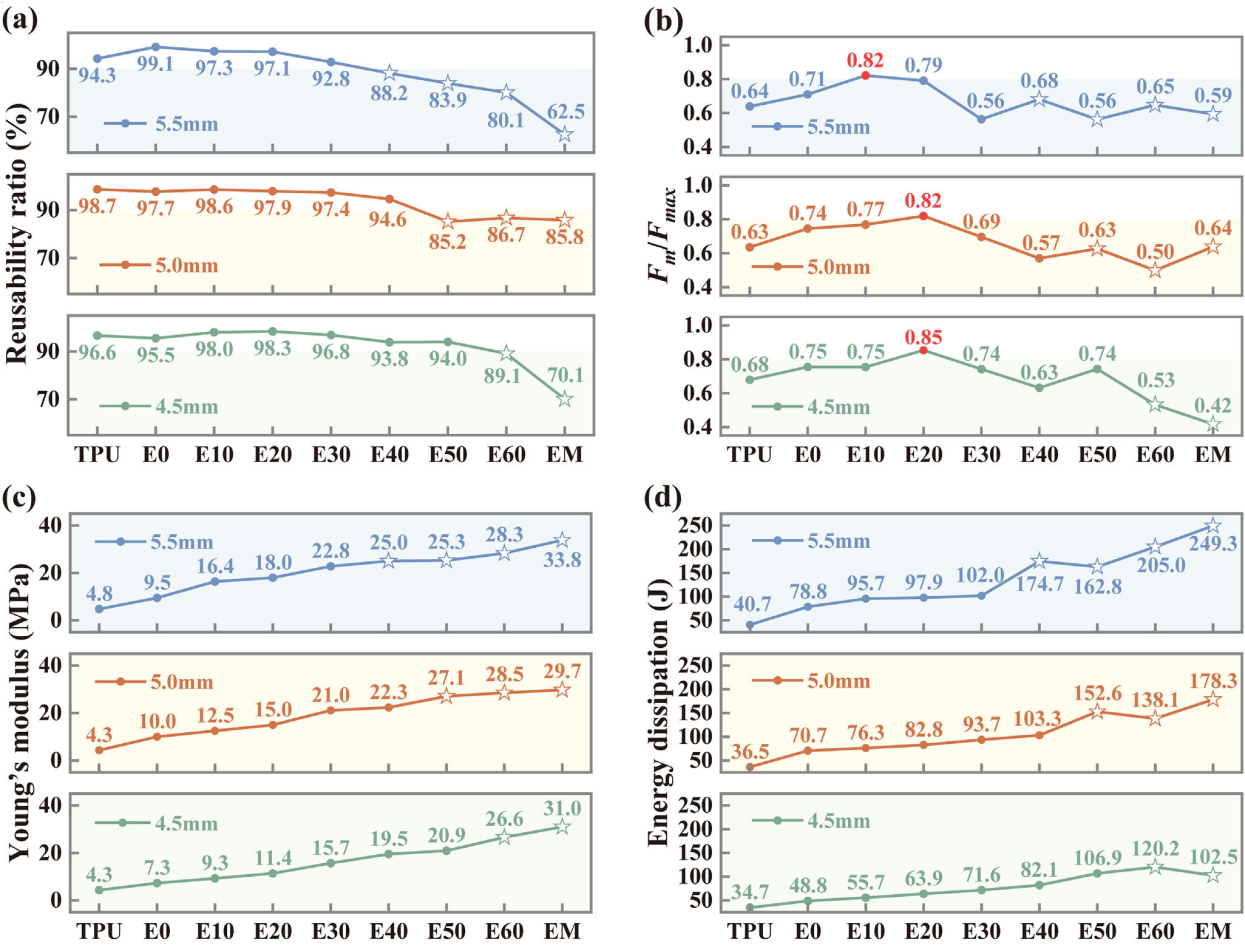
Key performance metrics of the LMPAO unit cell across a range of geometric parameters. (a) Reusability ratio. We consider structures with a reusability ratio lower than 90% to have lost reusability and mark the corresponding data points with hollow pentagrams. (b) Crushing force stability, defined as the ratio of the average crushing force *F_m_* to the maximum crushing force *F_max_* before the densification stage. A value closer to 1 indicates a more stable force response. (c) Equivalent Young's modulus of the metamaterial. (d) Energy dissipated in a single cycle.

To further elucidate the tunability of the mechanical response of the LMPAO unit cell from a quantitative perspective, Figure 3 summarizes key performance metrics of the unit cell across a range of structural parameters.

Figure 3a illustrates that the reusability ratio of the metamaterial drops sharply once the width of the metal skeleton exceeds a certain threshold, regardless of the structural thickness. We consider structures with a reusability ratio lower than 90% to have lost reusability and mark the corresponding data points with hollow pentagrams. This phenomenon can be attributed to the fact that as the metal skeleton becomes wider, its contact area with the TPU shell increases. The limited adhesion between the two materials weakens the structural integrity. During compression, partial separation occurs between the metal skeleton and the TPU shell, causing the deformation to deviate from the diamond mode. The TPU shell undergoes local deformation beyond its elastic limit, preventing full recovery and ultimately reducing the reusability ratio of the metamaterial.

Figure 3b presents the crushing force stability, characterized by the ratio *F_m_/F_max_*, where *F*_max_ and *F*_m_ denote the maximum and average crushing forces before the densification stage, respectively. At a thickness of 5.5 mm, the LMPAO with a metal skeleton of width level E10 exhibits the highest crushing force stability, while at 5.0 and 4.5 mm, those with a metal skeleton of width level E20 show the highest crushing force stability. In all three cases, the corresponding *F_m_/F_max_* values exceed 0.8.

Figure 3c presents the equivalent Young's modulus of the LMPAO for different geometric parameters. With the shell thickness fixed, increasing the skeleton width results in a higher equivalent Young's modulus, which indicates an increase in the overall structural stiffness. Figure 3d illustrates the energy dissipation of LMPAO structures in a single quasi‐static cycle. Within the reusability range, LMPAO structures with wider metal skeletons and larger thickness dissipate more energy. Figure 3c,d shows that, within the design space defined by the skeleton width and shell thickness, the equivalent stiffness and energy dissipation capability of the LMPAO exhibit broad tunability.

In summary, the mechanical response of the LMPAO unit cell exhibits multidimensional tunability. Adjusting the geometric parameters enables the tailoring of mechanical properties, including stiffness, buckling threshold, force plateau height, energy dissipation capability, rebound energy, and crushing force stability.

### 2.3 Performance Advantages of the Diamond Deformation Mode in the LMPAO

Previous studies have demonstrated that the diamond deformation mode imparts superior energy absorption characteristics to diamond origami metamaterials fabricated from metal [24, 25, 55]. However, the configuration and material system of the LMPAO differ significantly from those of traditional metal origami structures. Whether the diamond deformation mode still retains its advantage in the LMPAO remains unclear. We therefore fix the width level of the metal skeleton at E20 and vary the origami dihedral angle *θ* from 122° to 150° to construct a series of LMPAO unit cells with different dihedral angles. Based on these samples, we perform cyclic tests to elucidate the effect of the dihedral angle on the deformation mode and compare the mechanical responses under different deformation modes (see the raw experimental data in Section S8).

**FIGURE 4:**
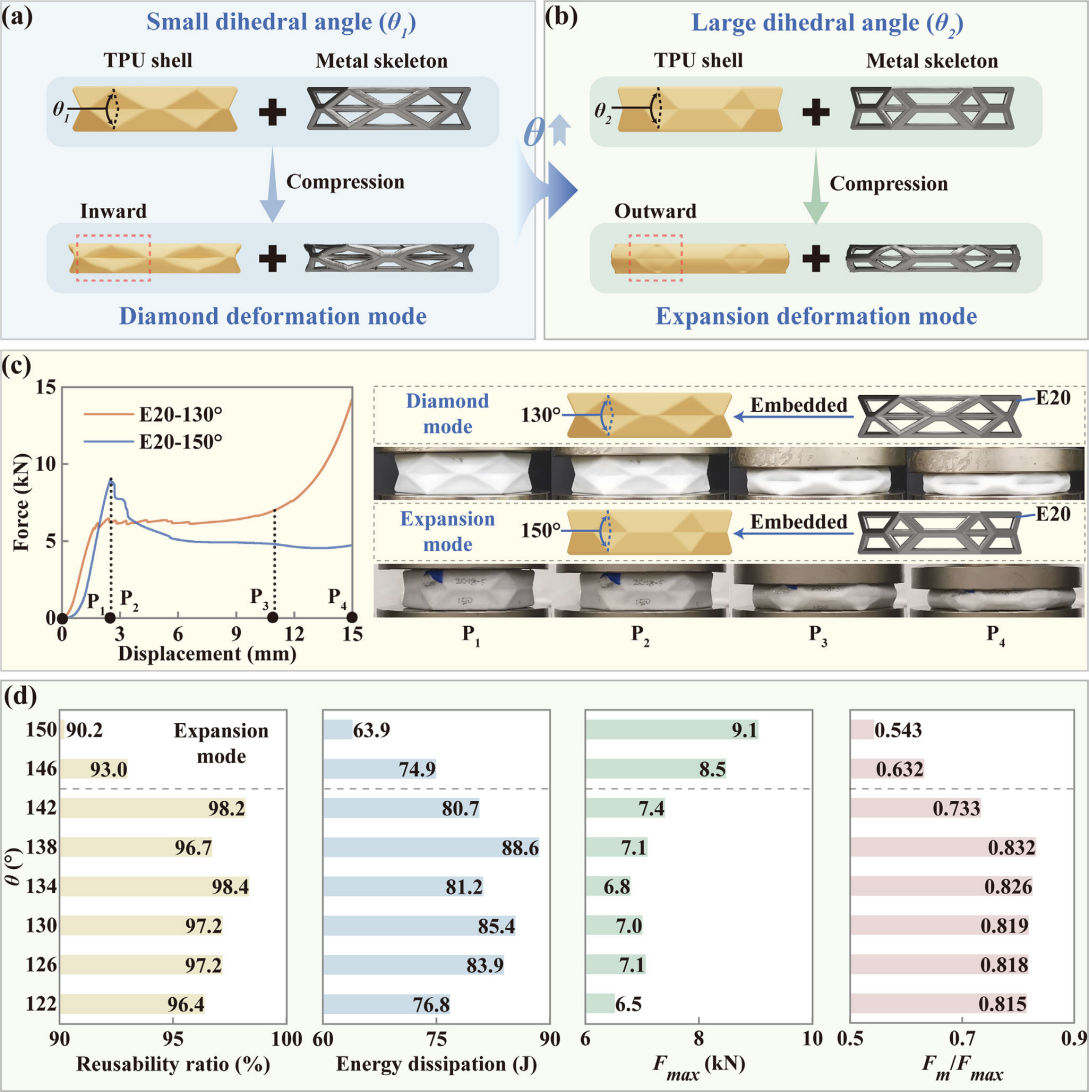
Mechanical responses of LMPAO with different dihedral angles. (a) Diamond deformation mode at small dihedral angles. (b) Expansion deformation mode at large dihedral angles. (c) Quasi‐static compression force–displacement curves of E20‐130° and E20‐150°, along with experimental snapshots at key deformation stages. (d) Reusability ratio, energy dissipation per cycle, maximum crushing force *F_max_* before densification, and crushing force stability *F_m_/F_max_* of the LMPAO with different dihedral angles.

At a small dihedral angle, the deformation of the TPU shell and the metal skeleton is consistently guided by the pre‐folded pattern, as shown in Figure 4a. In this case, the rhombic regions collapse inward while the trapezoidal regions expand outward. This deformation mode is referred to as the diamond mode. At a large dihedral angle, the rhombic regions of the TPU shell and the metal skeleton gradually bulge outward, as shown in Figure 4b. The deformation mode transforms into the expansion mode. In Figure 4c, E20‐130° and E20‐150° are selected as representative structures for the diamond and expansion modes, respectively, and their force–displacement curves together with experimental snapshots at key points are presented. The force–displacement curve corresponding to the diamond mode shows no IPCF and thus exhibits high crushing force stability, which is desirable for an ideal energy absorption curve. In contrast, the force–displacement curve corresponding to the expansion deformation mode presents a pronounced IPCF and a substantially reduced load level in the plateau stage, leading to a weakened energy absorption capacity (see the deformation process in Movie S2).

To quantitatively evaluate the effect of the dihedral angle, Figure 4d presents key performance metrics of LMPAO unit cells with different dihedral angles. The structural deformation exhibits the diamond mode at dihedral angles ranging from 122° to 138°, the expansion mode at 146° and 150°, and a transitional mode at 142°. The results show that the reusability ratio of the structure in the diamond mode is significantly higher than that in the expansion mode. This is because the local deformation of the TPU shell in the expansion mode exceeds its recoverable limit, resulting in impaired shape recovery after cycling. Meanwhile, the energy dissipation per cycle in the diamond mode is also significantly higher than that in the expansion mode. In addition, since the force–displacement curve corresponding to the diamond deformation mode exhibits no pronounced IPCF, the maximum force *F_max_* before densification remains relatively low, which consequently keeps the index *F_m_*/*F_max_* in a high range.

Overall, the diamond deformation mode still maintains its advantage in LMPAO. In the diamond mode, the structure exhibits high reusability, large energy dissipation per cycle, and high crushing force stability, whereas the expansion mode shows markedly inferior performance.

### 2.4 Optimization Strategies for Enhancing the Deformation Mode Stability of Multilayer LMPAO

**FIGURE 5:**
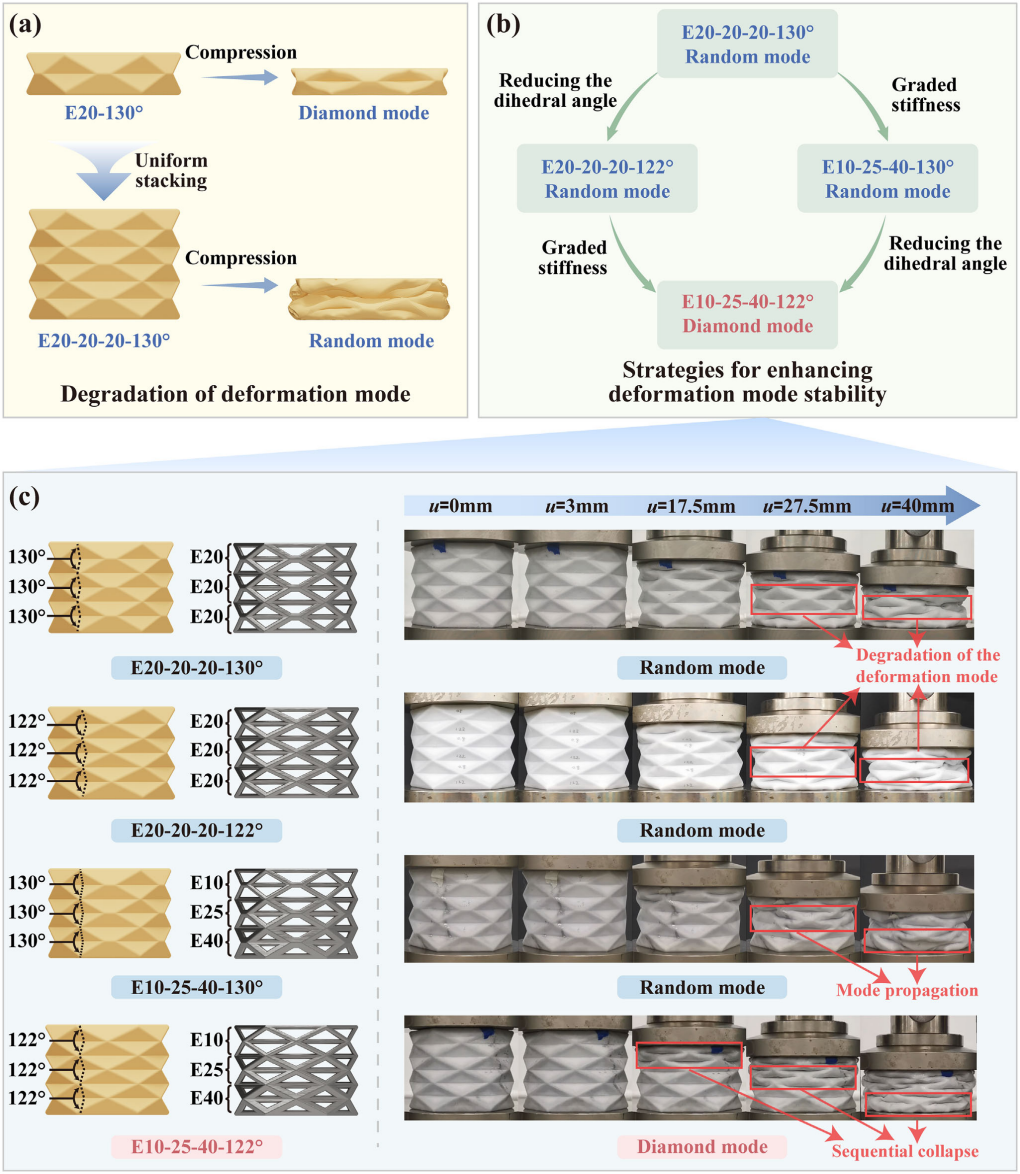
Optimization strategies for enhancing the deformation mode stability of the monolithic multilayer LMPAO. (a) Schematic illustration of the degradation of the deformation mode caused by uniform multilayer stacking. (b) Optimization paths for enhancing deformation mode stability of monolithic multilayer LMPAO. (c) Experimental snapshots of the compression deformation processes of monolithic multilayer LMPAO. The four configurations displayed here correspond to the configuration names listed in Figure 5b.

Considering that impact typically occurs over a limited contact area, it is crucial to enhance the energy absorption per unit projected area (*EA*/*A*) of the metamaterial. A common approach is to extend the compression stroke by axially stacking multiple unit cells. However, such axial stacking cannot be extended indefinitely. As shown in Figure 5a, once the number of uniformly stacked unit cells exceeds a certain threshold, the structure no longer preserves the diamond deformation mode during compression but instead degrades into a random mode. This random deformation mode would introduce randomness into the force–displacement curves during cyclic loading, thereby severely compromising structural reusability. Therefore, enhancing the deformation mode stability of multilayer LMPAO is crucial. This section presents three structural optimization strategies to address this issue.

#### 2.4.1 Dihedral Angle Reduction and Graded Stiffness Strategies

Using 3D printing technology, the monolithic multilayer LMPAO shell can be fabricated, thereby enabling the axial stacking of multiple unit cells. We extend the structure to three layers, and the quasi‐static loading process of E20‐20‐20‐130° is shown in the first row of Figure 5c. Each of the three layers has the same metal skeleton width and dihedral angle. During quasi‐static compression, the first layer collapses first, followed by the third layer. Eventually, the deformation of the second layer deviates from the guidance of the pre‐folded pattern and deteriorates from the diamond mode into a random mode. The primary cause of this phenomenon is the deformation coupling effect between the second layer and its adjacent layers (first and third) during compression. This coupling causes the loading direction of the second layer to deviate from the vertical direction and results in an inclined loading state. In this state, the pre‐folded pattern provides only limited guidance for the deformation mode and cannot resist the interference of fabrication‐induced microdefects, causing the deformation and force response of the structure to become random. This randomness is an undesirable feature that must be avoided in the design of energy‐absorbing structures.

We propose two preliminary optimization strategies to induce the overall structural deformation into a stable diamond mode. The first strategy is to reduce the dihedral angle, since smaller dihedral angles enable the pre‐folded pattern to provide a stronger inducing effect on the deformation mode. Accordingly, we set the dihedral angle of all three layers to 122° while keeping the width of the metal skeletons unchanged, yielding the E20‐20‐20‐122° configuration shown in the second row of Figure 5c. However, its deformation mode resembles that of the E20‐20‐20‐130°. The deformation coupling effect between structural layers causes a degradation of the deformation mode in the second layer, indicating that the optimization strategy shows limited effectiveness.

The second strategy is to introduce a graded stiffness design in the structure. During the compression of both the E20‐20‐20‐130° and E20‐20‐20‐122°, we observe that the deformation coupling between the second layer and the adjacent layers serves as the primary cause of the degradation of the deformation mode. As discussed in Section 2.2, we can adjust the stiffness of an LMPAO unit cell by varying the width of the metal skeleton, thereby controlling its buckling threshold. In axially stacked structures, unit cells with lower buckling thresholds collapse earlier in the deformation process. Therefore, a graded stiffness design can be implemented to introduce a gradient in the buckling thresholds across layers, ensuring that the unit cells collapse sequentially from the lowest to the highest buckling threshold. This orderly collapse ensures that the deformation at the interface between the second and third layers can be neglected during the collapse of the second layer. Only the deformation coupling between the first and second layers affects the stability of the deformation mode. Based on this strategy, we design the E10‐25‐40‐130° configuration shown in the third row of Figure 5c. During quasi‐static compression, the low‐stiffness first layer collapses first, followed by the medium‐stiffness second layer. However, the deformation of the second layer is not induced into the diamond mode but instead degrades into a random mode, which subsequently propagates into the third layer. Therefore, this graded stiffness optimization strategy shows limited effectiveness.

Each optimization strategy exhibits limited effectiveness when applied individually, and the deformation of the modified monolithic multilayer LMPAO does not exhibit a stable diamond deformation mode. Therefore, we further implement both optimization strategies simultaneously, namely reducing the dihedral angle while introducing a gradient in the width of the embedded metal skeleton. E10‐25‐40‐122° is the optimized structure, and the experimental snapshots of the key stages during its compression process are shown in the fourth row of Figure 5c. As the displacement increases, the first and second layers of the structure collapse sequentially. Notably, the deformation of each layer follows a stable and predictable diamond mode, and the optimized structure exhibits excellent reusability (see the mechanical responses of the structures before and after optimization in Section S9 and the comparative compression deformation processes in Movie S3).

Figure 5b provides an overview of how the two optimization strategies influence the deformation mode stability of the monolithic multilayer LMPAO. When the two strategies are implemented independently, the deformation mode of the multilayer LMPAO degrades. In contrast, simultaneously implementing both optimization strategies allows the pre‐folded pattern to effectively induce the structural deformation into the diamond mode. By controlling a single variable (E20‐20‐20‐122° → E10‐25‐40‐122° and E10‐25‐40‐130° → E10‐25‐40‐122°), we conclude that both reducing the dihedral angle and introducing a graded stiffness design can independently enhance the stability of the deformation mode of the monolithic multilayer LMPAO metamaterial.

#### 2.4.2 Deformation Coupling Elimination Strategy between Structural Layers

**FIGURE 6:**
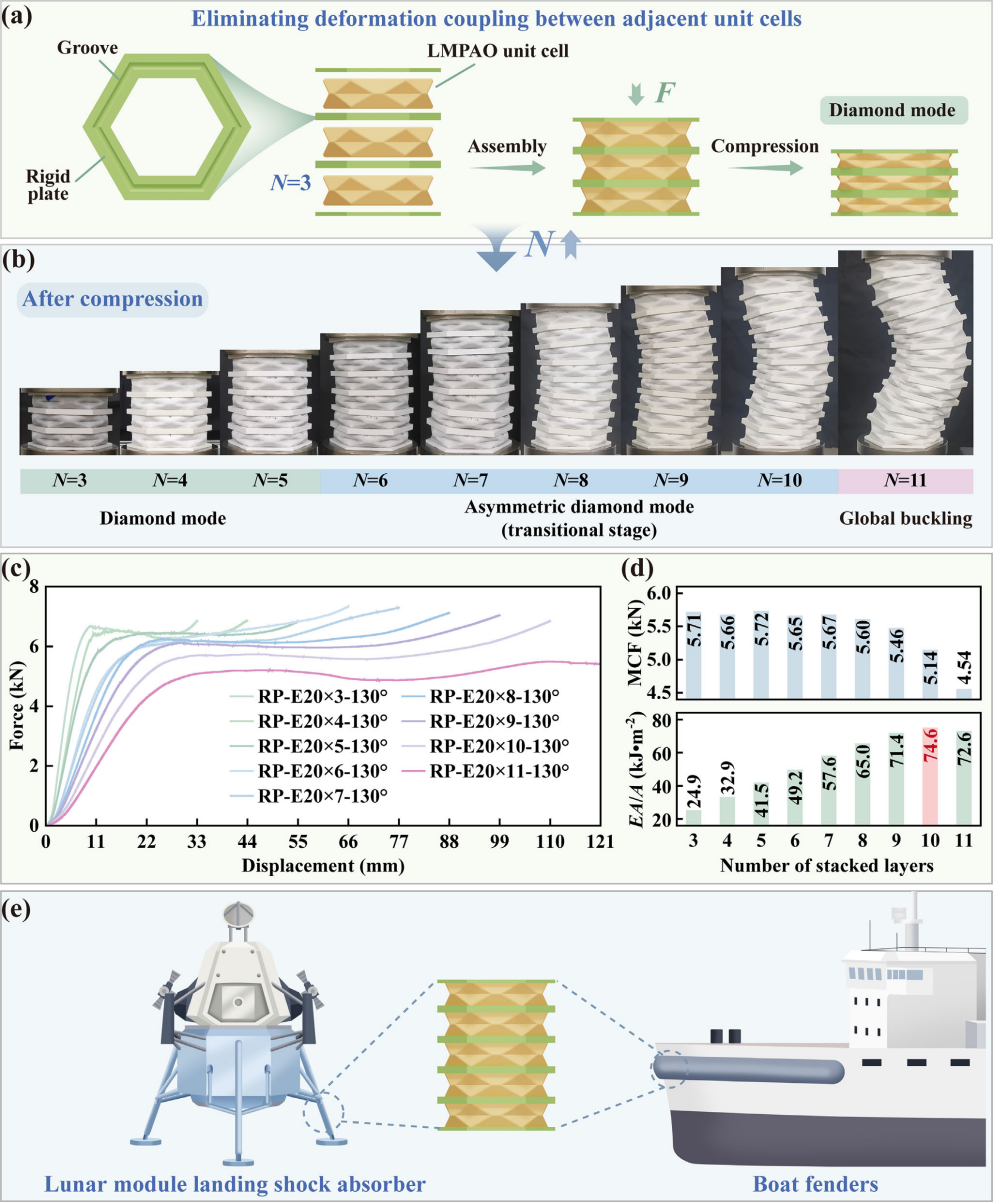
Deformation coupling elimination strategy between structural layers. (a) Schematic illustration of the optimization strategy. Rigid plates with grooves are used to connect and constrain the LMPAO unit cells to eliminate the deformation coupling between structural layers. (b) Post‐compression states of modular LMPAO structures with different numbers of layers. (c) Force–displacement curves of modular LMPAO structures with different numbers of layers. (d) MCF and *EA*/*A* of modular LMPAO structures with different numbers of layers. (e) Examples of potential engineering applications.

As revealed in Section 2.4.1, the deformation coupling between structural layers is the primary cause of the degradation of the deformation mode in multilayer LMPAO structures. To address this issue, this section introduces an optimization strategy for eliminating deformation coupling between structural layers. As shown in Figure 6a, we first fabricate multiple LMPAO unit cells and then axially connect and constrain them using rigid plates with grooves, thereby obtaining a modular multilayer LMPAO metamaterial (see the geometric details and fabrication method of the plates in Section S10). This assembly method is designed to make the deformation of each layer in the multilayer LMPAO relatively independent during compression, thereby helping the overall structure maintain the diamond deformation mode.

To verify the effectiveness of the above modular assembly strategy in enhancing the deformation mode stability of multilayer LMPAO, we select the E20‐130° unit cell, which produces an ideal energy absorption curve, as the base configuration. We then fabricate modular LMPAO structures with 3 to 11 stacked layers and conduct quasi‐static compression tests (see the complete compression processes and raw data in Sections S11 and S12).

Figure 6b shows the post‐compression states of modular LMPAO structures with different numbers of stacked layers. As the number of stacked layers increases, the structural deformation mode shifts from the diamond mode to the asymmetric diamond mode and finally to the global buckling mode. In contrast to the random deformation mode observed in E20‐20‐20‐130° (a monolithic three‐layer structure) in Section 2.4.1, the modular LMPAO maintains the diamond deformation mode well for 3–5 stacked layers. The overall deformation remains relatively uniform, with no obvious lateral offset. As the number of stacked layers further increases to 6–10, the structure begins to show a slight lateral offset after compression, and the offset becomes more pronounced with increasing layer number. Although the deformation of each layer is still induced by the pre‐folded pattern and retains characteristics of diamond folding, the unit‐cell deformation is no longer fully uniform or symmetric. Therefore, we define this deformation mode as the asymmetric diamond mode and regard it as a transitional stage between the diamond mode and global buckling. When the number of stacked layers is further increased to 11, the overall structure shows a significant lateral offset after compression, and the deformation on the two sides of the unit cells becomes highly uneven. For example, in the middle‐layer unit cell, the left side has already entered the densification stage, whereas the right side shows only slight deformation. In this case, the structural deformation mode has degraded from the asymmetric diamond mode to global buckling.

Figure 6c shows the force–displacement curves of modular LMPAO structures with different numbers of layers. The curves for all structures exhibit high crushing force stability, a long plateau stage, and minimal fluctuations, representing ideal energy absorption profiles. In addition, the force plateau height remains nearly constant for the 3–10‐layer structures. A pronounced decrease in the plateau height occurs only when the structure is stacked to 11 layers, where the deformation mode has further degraded into global buckling.

Figure 6d presents the mean crushing force (MCF) and energy absorption per unit projected area (*EA*/*A*) of modular LMPAO structures with different numbers of stacked layers. As the number of stacked layers increases, the MCF initially remains nearly constant and then gradually decreases. When the layer number reaches 11, global buckling occurs, causing the MCF to drop sharply to 4.54 kN. *EA*/*A* shows a different trend. It increases first and then decreases. *EA*/*A* reaches a maximum of 74.59 kJ·m^−2^ at 10 layers and then decreases to 72.55 kJ·m^−2^ when the number of layers increases to 11. This result indicates that increasing the number of stacked unit cells can improve *EA*/*A* by extending the effective compression stroke. However, once global buckling occurs, the MCF drops sharply. In this case, the additional compression stroke can no longer offset the negative effect of the reduced MCF on total energy absorption, leading to a decrease in *EA*/*A*.

Collectively, the optimization strategy that eliminates deformation coupling between structural layers significantly improves the stability of the deformation mode in multilayer LMPAO structures. It extends the effective compression stroke of the structure, thereby increasing its energy absorption per unit projected area. However, this increase is not unlimited. Excessive axial stacking of unit cells will induce global buckling, which instead leads to a decrease in *EA*/*A*. It should be noted that this strategy inevitably introduces additional mass, volume, projected area, and assembly complexity. Therefore, its use should be carefully weighed based on the specific optimization metrics. According to the calculations, the structure (with five stacked layers) achieves an energy absorption per unit projected area of up to 41.5 kJ·m^−^^2^ in a single cycle, exhibits a crushing force stability of 0.838, and maintains a reusability ratio of 97.8%, demonstrating a synergistic combination of high‐quality energy absorption and high reusability. With this combination of properties, the structure shows great potential for engineering applications that require repeated energy absorption, such as the lunar module landing shock absorber and boat fenders shown in Figure 6e.

## 3 Discussion and Conclusions

The primary advantage of the proposed LMPAO metamaterial is its ability to achieve high reusability and high‐quality energy absorption simultaneously. Here, the energy absorption quality encompasses two aspects: energy absorption capacity and crushing force stability. Energy absorption capacity can be quantitatively characterized using various methods. Energy absorption per unit volume (*EA*/*V*) is one widely used indicator. Considering that impact usually occurs over a limited contact area, energy absorption per unit projected area (*EA*/*A*) is also included in the performance comparison [35]. Here, *EA* denotes the energy absorbed by the structure before densification, *V* denotes the structural volume, and *A* denotes the projected area of the structure (see the detailed definitions of *A* and *V* in Section S13).

**FIGURE 7:**
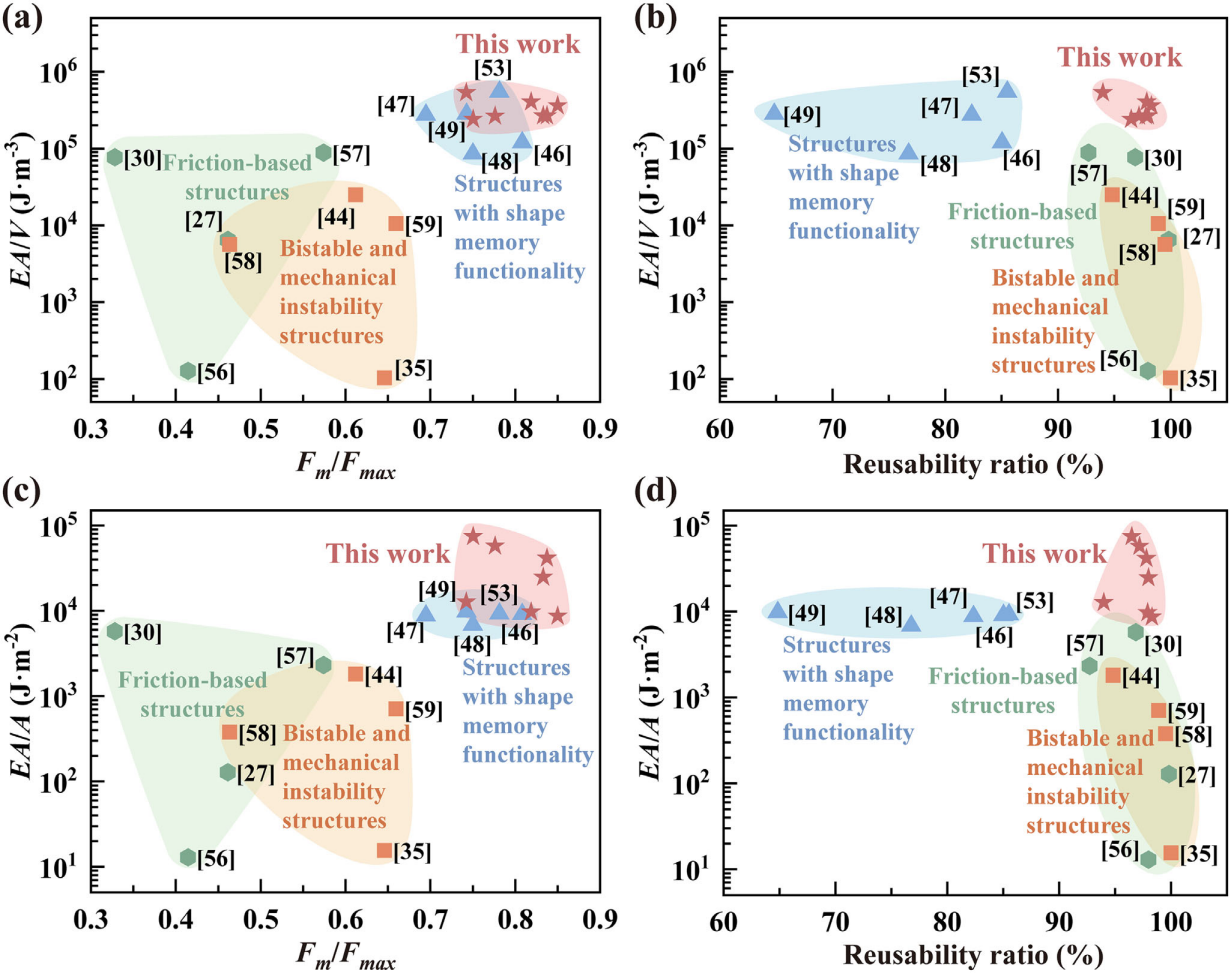
Performance comparison between the proposed metamaterial and existing reusable energy‐absorbing metamaterials. Currently, reusable energy‐absorbing metamaterials typically include friction‐based structures [27, 30, 56, 57], structures based on bistability or mechanical instability [35, 44, 58, 59], and structures with shape memory functionality [46, 47, 48, 49, 53]. (a) Comparison in terms of *EA*/*V* and crushing force stability. (b) Comparison in terms of *EA*/*V* and reusability. (c) Comparison in terms of *EA*/*A* and crushing force stability. (d) Comparison in terms of *EA*/*A* and reusability.

We present four Ashby plots in Figure 7 to provide a comprehensive comparison of the overall performance of various reusable energy‐absorbing metamaterials. As shown in Figure 7b,d, friction‐based structures and structures based on bistability or mechanical instability exhibit outstanding reusability. However, Figure 7a,c shows that their energy absorption quality remains significantly limited. In friction‐based structures, the presence of stick‐slip friction may introduce severe oscillations in the force–displacement curve, resulting in low crushing force stability [27, 30, 56, 57]. Structures based on bistability or mechanical instability are greatly limited in their energy absorption capacity due to the low stiffness of the constituent materials [35, 44, 58, 59].

In contrast, structures based on shape memory alloys (SMA) and shape memory polymers (SMP) dissipate energy through hysteretic behavior associated with phase transformation and viscoelasticity, respectively, and their energy absorption capacity is improved to some extent [46, 47, 48, 49]. Moreover, because their structural configurations closely resemble those of conventional energy absorbers designed for one‐time usage, the crushing force stability can be optimized through standard structural design approaches. Figure 7a,c show that this type of structure achieves excellent performance in both energy absorption capacity and crushing force stability, reflecting a high overall energy absorption quality. However, due to the damage accumulation that occurs in structures based on SMP and SMA during cyclic loading, their reusability is substantially constrained, as shown in Figure 7b,d.

The proposed LMPAO simultaneously exhibits high energy absorption quality and high reusability. As shown in Figure 7a,b, when *EA*/*V* is used to characterize energy absorption capacity, the LMPAO achieves an *EA*/*V* of 404.8 kJ·m^−^^3^ while maintaining a crushing force stability of 0.819 and a reusability ratio of 97.9%. As shown in Figure 7c,d, when *EA*/*A* is used to characterize energy absorption capacity, the LMPAO achieves an *EA*/*A* of 41.5 kJ·m^−^^2^ while maintaining a crushing force stability of 0.838 and a reusability ratio of 97.8%. Under both evaluation systems, the LMPAO shows energy absorption quality comparable to that of structures based on SMA and SMP. Meanwhile, in terms of reusability, this metamaterial exhibits performance comparable to that of friction‐based structures and structures based on bistability or mechanical instability. These results demonstrate that the proposed LMPAO breaks the trade‐off between high energy absorption quality and high reusability in existing reusable energy‐absorbing metamaterials.

The exceptional overall performance of the proposed LMPAO stems from its synergistic design at the material, unit‐cell configuration, and multi‐cell system levels. At the material level, the LMPAO achieves exceptional energy absorption through the plastic deformation of its metal skeleton. This plastic deformation mechanism enables its energy absorption capacity to far exceed that of structures based on bistability or mechanical instability. In addition, the heat‐induced solid–liquid phase transition of the LMPA eliminates its previously plastically deformed state. On this basis, the hyperelasticity of the TPU shell provides a sufficient driving force for structural recovery, thereby endowing the metamaterial with superior reusability compared to structures based on SMP and SMA. At the unit‐cell configuration level, adjusting the width of the metal skeleton enables the LMPAO unit cell to achieve exceptionally high crushing force stability. Moreover, the adoption of the diamond origami pattern ensures that the structural deformation follows a predictable diamond mode, which facilitates the simultaneous improvement of its crushing force stability and energy absorption capacity. At the multi‐cell system level, the optimization strategy that eliminates deformation coupling between structural layers significantly enhances the stability of the deformation mode in multilayer structures. This enhancement extends the upper limit of the effective compression stroke, contributing to increased energy absorption per unit projected area of the structure. More importantly, the designs across these three levels do not impose mechanistic conflicts, ensuring that improvements in one performance dimension do not compromise another.

## 4 Experimental Section

### 4.1 Design and Fabrication

The LMPAO unit cell consists of a diamond origami elastomeric shell with hollow channels in the crease regions and an LMPA skeleton embedded in these channels. The geometric dimensions are provided in Sections S1–S3. The diamond origami elastomeric shell was fabricated using a Bambu Lab A1 3D printer based on fused deposition modeling (FDM) technology. A commercial TPU filament (eSUN thermoplastic polyurethane) with a Shore hardness of 95A was selected as the printing material for FDM 3D fabrication. The LMPA used in this study was an InSnBi alloy with a melting point of 47°C. We heated the LMPA to 60°C until it melted and subsequently injected it into the TPU shell through pre‐reserved small holes using a syringe. As the final step, we left the structure to cool down, thus enabling the molten metal to solidify and form the LMPA skeleton, which was seamlessly and cleverly embedded into the TPU shell. In Section 2.4.1, we fabricated the monolithic multilayer LMPAO using the same method as that for the unit cell. In Section 2.4.2, we constructed the modular multilayer structure by axially connecting and constraining unit cells using rigid plates with grooves. We fabricated the rigid plates using FDM 3D printing, with commercial PLA filament (eSUN polylactic acid) as the printing material. The detailed geometric dimensions are provided in Section S10.

### 4.2 Finite Element Simulations

We conducted quasi‐static simulations of the compressive behavior of the LMPAO using the commercial finite element software ABAQUS/Explicit. In the material definition stage, we assigned an elastoplastic constitutive model to the LMPA skeleton and a hyperelastic constitutive model to the TPU shell. Both materials were meshed using C3D10M solid elements. During loading, the LMPAO was placed between two rigid plates, with the bottom plate fixed and the top plate moving downward to apply compression. The friction coefficient for tangential contact between the rigid plates and the LMPAO was set to 0.3, and we applied a tie constraint between the LMPA skeleton and the TPU shell. To reduce the computational time in the explicit analysis, we appropriately increased the loading speed. As long as the kinetic energy remained within 5% of the internal energy, the accuracy of the simulation results could be ensured.

### 4.3 Quasi‐Static Compression‐Unloading Test and Recovery Process of the LMPAO

We conducted quasi‐static compression‐unloading tests on the LMPAO at room temperature (20°C) using a universal testing machine (Zwick Roell Z050). The LMPAO was placed between two compression platens, with the lower platen fixed and the upper platen moved downward at a speed of 10 mm/min. The test entered the quasi‐static unloading stage once it reached the maximum preset displacement. During this stage, the unloading speed remained constant, and the test ended when the force measured by the sensor dropped to zero. Heating the tested sample in a 60°C oven for 10 min triggered the shape recovery of the LMPAO, and then the sample was cooled at room temperature for 30 min. After the metal skeleton fully solidified, the LMPAO achieved full recovery, marking the end of one test cycle. Repeating the above procedure for multiple cycles allowed us to evaluate the reusability of the LMPAO. To control a single variable, we also heated and cooled the tested sample before the first cycle.

## Author Contributions

**Yupeng Liu**: conceptualization, methodology, software, investigation, validation, formal analysis, visualization, writing – original draft, data curation, writing – review and editing. **Wei Zhao**: project administration, resources, supervision, writing – review and editing, funding acquisition. **Chengjun Zeng**: writing – review and editing, project administration. **Yanju Liu**: resources, project administration, funding acquisition. **Jiuming Fan**: supervision, writing – review and editing, project administration. **Jinsong Leng**: resources, project administration, funding acquisition.

## Funding

National Natural Science Foundation of China (Grant No. 92271206)

## Conflicts of Interest

The authors declare no conflicts of interest.

## Supporting information




**Supporting File 1**: advs76536‐sup‐0001‐SuppMat.pdf.

**Supporting File 2**: advs76536‐sup‐0002‐MovieS1.mp4.

**Supporting File 3**: advs76536‐sup‐0003‐MovieS2.mp4.

**Supporting File 4**: advs76536‐sup‐0004‐MovieS3.mp4.

## Data Availability

The data that support the findings of this study are available from the corresponding author upon reasonable request.
