# Supplementary material for: Origami Metamaterials Based on Low‐Melting‐Point Alloy Phase Transition: Breaking the Trade‐Off Between Reusability and Energy Absorption Quality
Source: Adv Sci (Weinh). 2026 Jul 9:e76536. Online ahead of print. doi: 10.1002/advs.76536 (PMC13348649; doi:10.1002/advs.76536)
Supplement: Supplementary file 1 — Supporting File 1: advs76536‐sup‐0001‐SuppMat.pdf. [file ADVS-9999-e76536-s003.pdf]

Supporting Information

Origami Metamaterials Based on Low-Melting-Point Alloy Phase Transition: Breaking the Trade-Off between Reusability and Energy Absorption Quality

Yupeng Liu, Wei Zhao*, Chengjun Zeng*, Jiuming Fan, Yanju Liu*, Jinsong Leng

Y. Liu (Yupeng Liu), W. Zhao, C. Zeng, J. Fan, Y. Liu (Yanju Liu)
Department of Astronautical Science and Mechanics, Harbin Institute of Technology (HIT), Harbin 150006, P. R. China.
E-mail: zhaowei_2022@163.com (Wei Zhao); zeng_cj@hit.edu.cn (Chengjun Zeng); yj_liu@hit.edu.cn (Yanju Liu)

J. Leng
Center for Composite Materials and Structures, Harbin Institute of Technology (HIT), Harbin 150080, P. R. China.

W. Zhao
Guangzhou Institute of Future Additive Manufacturing, Guangzhou 510360, P. R. China.

Y. Liu (Yanju Liu)
Suzhou Research Institute, Harbin Institute of Technology (HIT), Suzhou 215100, P. R. China.

S1: Geometric Parameter Relationships of the Diamond Origami
S2: Modeling Method of the LMPAO
S3: LMPAO Configurations for Investigating the Effects of Skeleton Width and Structure Thickness on Mechanical Response
S4: Indicators for Evaluating Energy Absorption
S5: Force–Displacement Curves from Cyclic Tests at an LMPAO Thickness of 5 mm
S6: Force–Displacement Curves from Cyclic Tests at an LMPAO Thickness of 4.5 mm
S7: Force–Displacement Curves from Cyclic Tests at an LMPAO Thickness of 5.5 mm
S8: Force–Displacement Curves from Cyclic Tests of LMPAO with Different Dihedral Angles
S9: Mechanical responses of monolithic three-layer LMPAO before and after optimization
S10: Geometric Dimensions of the Rigid Plates
S11: Quasi-static compression process of modular multilayer LMPAO
S12: Force–displacement curves of modular multilayer LMPAO during compression
S13: Definitions of the normalized reference area A and volume V
S14: Tensile Samples and Engineering Stress–Strain Curves of TPU-95A and InSnBi Alloy
S15: Ashby plot using SEA to characterize energy absorption capacity
S16: Design of Dual-Plateau Modular LMPAO Metamaterials
S17: Recovery mechanisms of existing reusable energy-absorbing metamaterials
S18: Service life of the LMPAO metamaterial

S1: Geometric Parameter Relationships of the Diamond Origami

Figure S1a shows the crease pattern of the diamond origami in its unfolded state, where solid lines represent mountain creases and dashed lines represent valley creases. The area enclosed by the bold red solid lines represents one unit cell of the pattern, which consists of two vertically symmetric rhombuses and two isosceles trapezoids. The entire crease pattern contains six unit cells. The valley crease length c, the base length of the trapezoid b, and the vertical height L of the crease pattern uniquely determine the geometric parameters of the crease pattern. The pattern can be folded along the creases, and Figure S1b shows the folding process of the pattern within the gray dashed box in Figure S1a. The folding process is a single-degree-of-freedom motion and can be controlled by the dihedral angle θ or the base angle ϕ. The angles θ and ϕ always satisfy the geometric relationship shown below:

$$\varphi = \pi - 4\tan^{-1}\left(\frac{L}{c}\cos\left(\frac{\theta}{2}\right)\right) \tag{1}$$

When the crease pattern is folded to connect the ends, the zero-thickness diamond origami structure shown in Figure S1c is formed. At this point, the structure has zero degrees of freedom, and ϕ equals 120°. Therefore, Equation (1) degenerates to:

$$\theta = 2\cos^{-1}\left(\tan\left(\frac{\pi}{12}\right)\frac{c}{L}\right) \tag{2}$$

Obviously, a simple geometric relationship exists between the layer height h of the diamond origami and the vertical height L of the crease pattern:

$$h = L\sin\left(\frac{\theta}{2}\right) \tag{3}$$

Using Equations (2) and (3), the valley crease length c and the vertical height L of the crease pattern can be expressed in terms of the dihedral angle θ and the layer height h of the diamond origami structure:

$$c = \frac{h}{\tan\left(\frac{\theta}{2}\right)\tan\left(\frac{\pi}{12}\right)} \tag{4}$$

$$L = h\sin\left(\frac{\theta}{2}\right) \tag{5}$$

Therefore, the geometric dimensions of the diamond origami structure shown in Figure S1c can be controlled by three independent variables: the bottom edge length b, the dihedral angle θ, and the layer height h. We fix the bottom edge length b at 50 mm and the layer height h at 24 mm in this work; therefore, we use the dihedral angle θ as the sole parameter to characterize the geometric dimensions of the diamond origami structure.

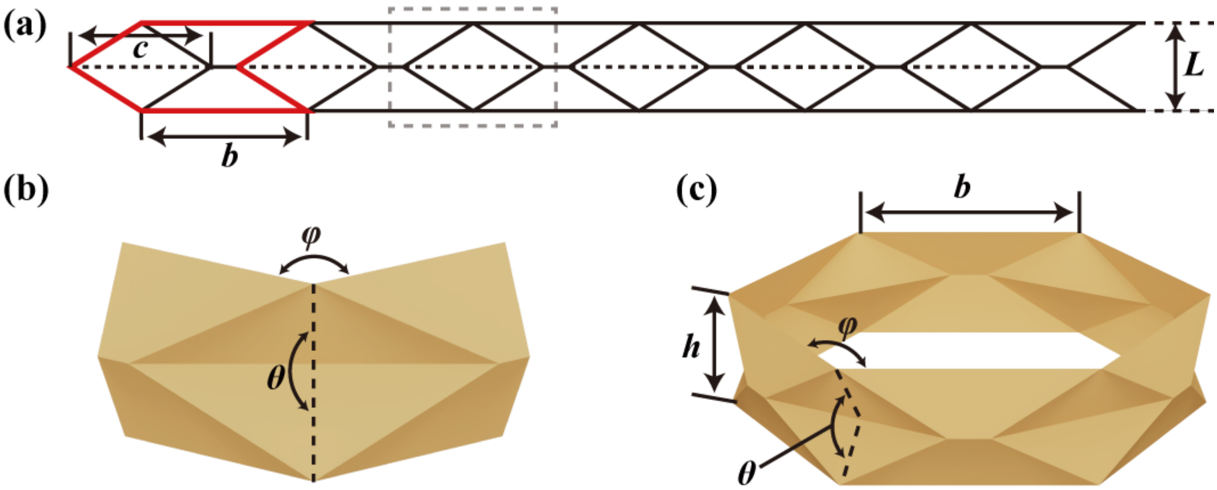

Figure S1. Zero-thickness diamond origami structure**.** a) Crease pattern. Solid lines represent mountain creases, and dashed lines represent valley creases. The region enclosed by the bold red solid lines denotes one unit cell, and the entire pattern contains six unit cells. b) Folding process of the crease pattern within the dashed box. Here, φ represents the angle between adjacent bottom edges. c) 3D schematic of the diamond origami structure.

S2: Modeling Method of the LMPAO

Based on the diamond origami modeling described in Section S1, we further develop a model of the LMPAO. Specifically, we first scale down the trapezoidal and triangular facets of the diamond origami structure shown in **Figure S2a** to α% of their original size, using their geometric centers as the scaling origins. Figure S2b illustrates the scaling process, where the green dashed lines represent the original size, the black solid lines denote the scaled-down size, and the gray-shaded area characterizes the degree of scaling, $\gamma\%$ ($\gamma\% = 1 - \alpha\%$). An original metal skeleton with a diameter of d is subsequently constructed along the creases of the diamond origami, as illustrated in Figure S2c. We then refer to the gray-shaded area in Figure S2b and expand the skeleton by $\gamma\%$ from the creases into the facets, forming the final metal skeleton model shown in Figure S2e. The metal skeleton has a thickness of d, and its width level is denoted by $E\gamma$.

To obtain the TPU shell model, we first assign a thickness t to the zero-thickness origami structure shown in Figure S2a, thereby generating the thickened origami model illustrated in Figure S2d. We then perform a Boolean subtraction between this model and the metal skeleton model shown in Figure S2e to obtain the TPU shell model illustrated in Figure S2f. Embedding the low-melting-point metal skeleton into the TPU shell yields the LMPAO shown in Figure S2g.

At this stage, the geometry of the LMPAO is governed by six independent variables: the

base length b, the dihedral angle θ, the layer height h, the metal skeleton thickness d, the width level of the metal skeleton $E\gamma$, and the structural thickness t. In this work, d and t satisfy the following relationship:

$$d = t - 2 \quad (6)$$

This ensures that the minimum thickness of the TPU shell remains 1 mm. In this study, the base length b is consistently set to 50 mm and the layer height h to 24 mm. Unless otherwise specified, the structural thickness t is 5 mm and the metal skeleton thickness d is 3 mm. Therefore, the geometric dimensions of the LMPAO are solely determined by the width level $E\gamma$ of the metal skeleton and the dihedral angle θ of the structure, and the structure is accordingly designated as $E\gamma$-θ.

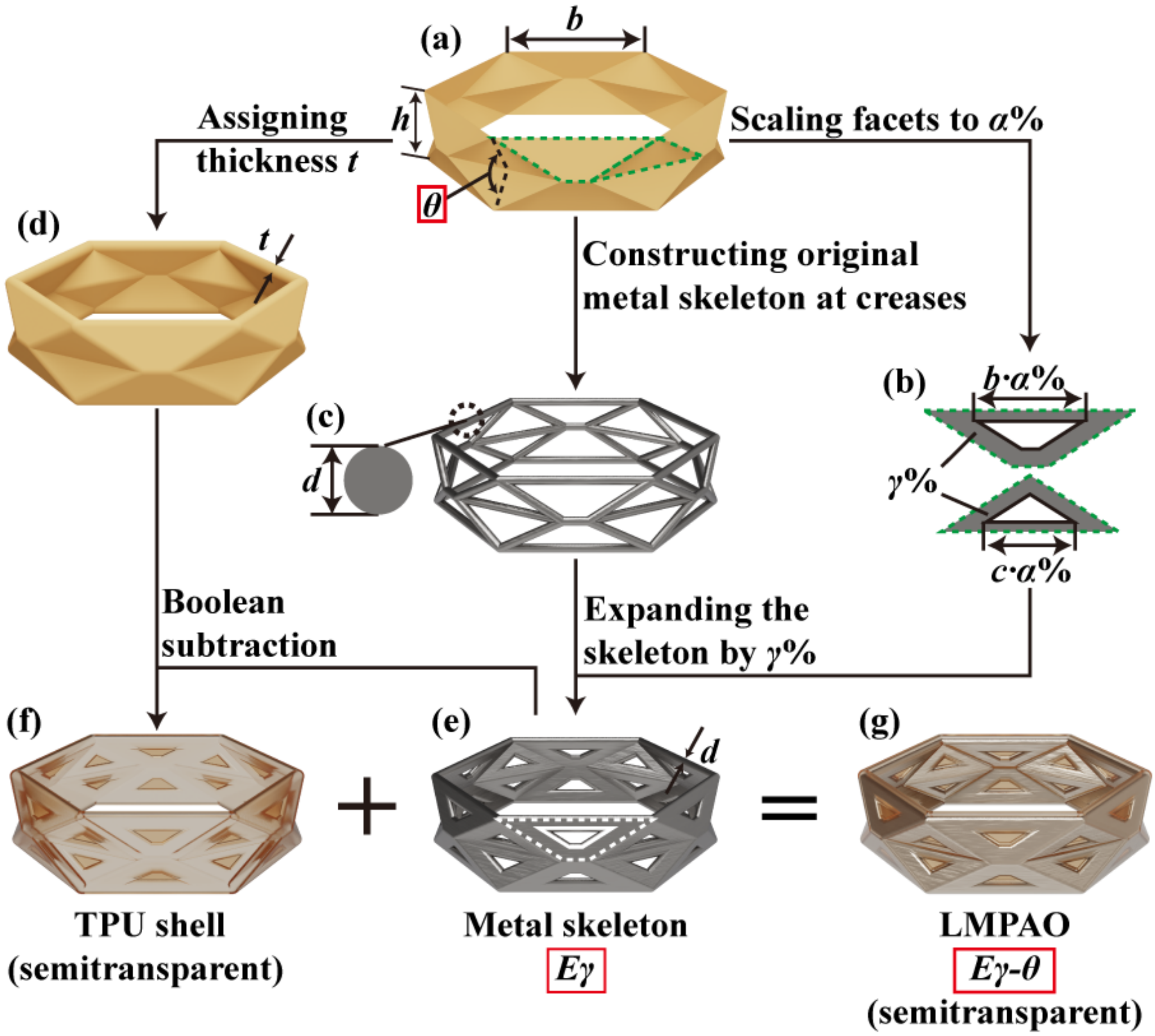

Figure S2. Modeling method of the LMPAO. a) Three-dimensional structure of the diamond origami with zero thickness. b) Schematic of facet scaling for constructing the metal skeleton. The trapezoidal and triangular facets of the origami structure are scaled down to α% of their original dimensions. The green dashed lines represent the original size, the black solid lines represent the scaled-down size, and the gray shaded regions indicate the degree of reduction, γ%, where $\gamma\% = 1 - \alpha\%$. c) Original metal skeleton model constructed along the creases of the

origami structure, with a diameter of *d*. d) Diamond origami model with assigned thickness *t*. e) Final metal skeleton model, formed by expanding the original metal skeleton inward into the facets by *γ*%; its width level is denoted by *Eγ*. f) TPU shell model. The semitransparent rendering clearly reveals the shell structure. g) LMPAO model, composed of the TPU shell and the embedded metal skeleton, denoted *Eγ*-*θ*.

S3: LMPAO Configurations for Investigating the Effects of Skeleton Width and Structure Thickness on Mechanical Response

Figure S3 illustrates the variations in LMPAO configurations associated with different width levels of the metal skeleton. By combining three structural thicknesses with eight widths of the metal skeleton, 24 LMPAO configurations are obtained. These structures are used to investigate the effects of metal skeleton width and structural thickness on the mechanical response of the LMPAO. It is worth noting that, for each thickness, a TPU origami structure without an embedded metal skeleton is required to serve as a control.

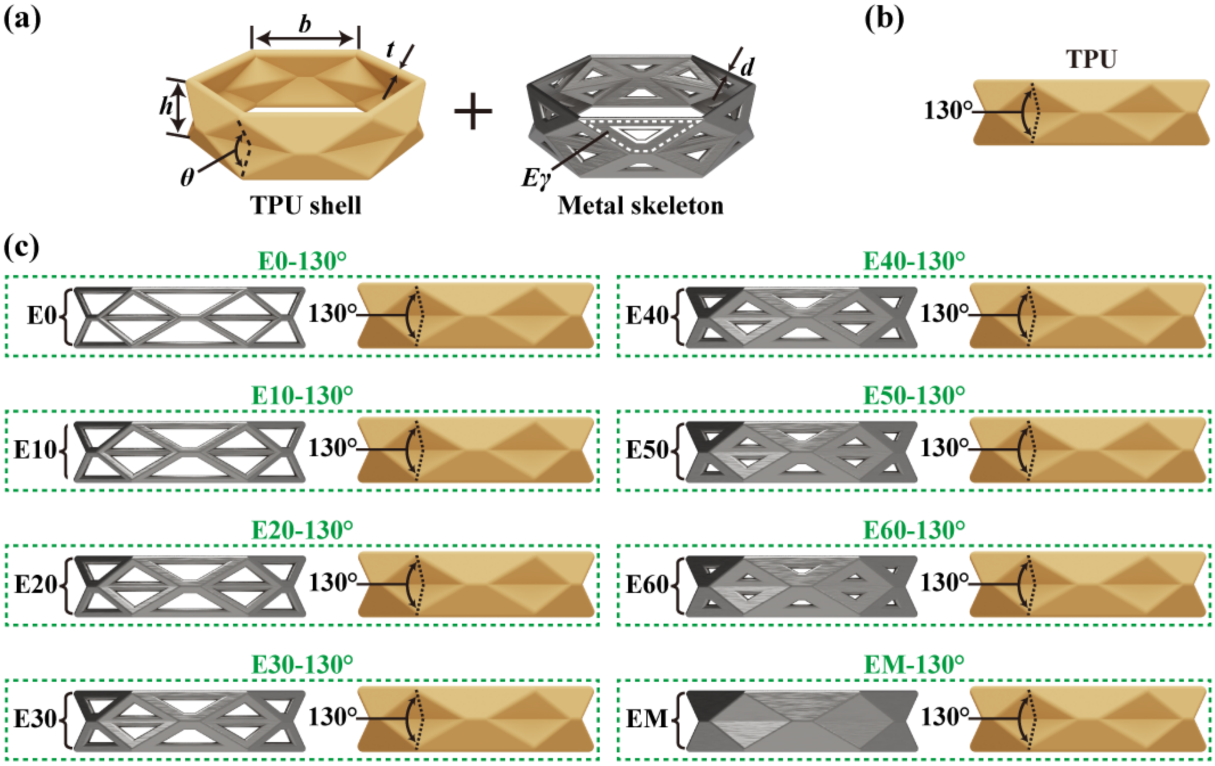

Figure S3. Structural schematic of the LMPAO. a) Composition of the LMPAO, with all geometric parameters kept constant except for the structural thickness *t* and the width level *Eγ* of the metal skeleton. b) TPU origami structure without an embedded metal skeleton, used as a control. c) Eight LMPAO structures embedded with metal skeletons of different widths.

S4: Indicators for Evaluating Energy Absorption

We define several quantitative indicators to evaluate the energy absorption of the LMPAO and illustrate them using representative force–displacement curves.

The rebound energy, indicated by the blue-shaded area in **Figure S4a**, is defined as the energy released by the LMPAO during the unloading process.

The energy dissipation is indicated by the green-shaded area in Figure S4a. It is defined as the energy absorbed by the LMPAO during compression minus the energy released during unloading.

Considering that impact usually occurs over a limited contact area, we define the energy dissipation per unit area (EDA) as an indicator to characterize the energy absorption capacity:

$$EDA = \frac{Energy\ dissipation}{A} \tag{7}$$

Where A denotes the contact area during impact.

The rebound ratio is defined as the ratio of rebound energy to the sum of rebound energy and energy dissipation:

$$Rebound\ ratio = \frac{Rebound\ energy}{Rebound\ energy + Energy\ dissipation} \tag{8}$$

The linear stage and the plateau stage of the force–displacement curve are regarded as the effective energy absorption stages. The corresponding displacement is defined as the effective stroke δ. We define the average crushing force within the effective stroke as F_m:

$$F_m = \frac{1}{\delta}\int_0^{\delta} F(s)ds \tag{9}$$

Where $F(s)$ is the displacement-dependent crushing force.

We define the maximum crushing force within the effective stroke as F_{max}. As illustrated in Figure S4a, for curves exhibiting an initial peak crushing force (IPCF), F_{max} typically corresponds to the IPCF. As illustrated in Figure S4b, for the curves without an initial peak crushing force (IPCF), F_{max} typically corresponds to the crushing force at the onset of the densification stage.

F_m/F_{max} can be used to evaluate the stability of the crushing force, and a value closer to 1 indicates a higher degree of stability.

The average crushing force $\overline{F}_m$ in the plateau stage is given by Equation (10):

$$\overline{F}_m = \frac{\int_{\tau}^{\delta} F(s)ds}{\delta - \tau} \tag{10}$$

Here, τ denotes the displacement corresponding to the onset of the plateau stage (P2).

The plateau force fluctuation quantifies the variation in crushing force during the plateau stage of the force–displacement curve and is defined as:

$$\text{Plateau force fluctuation} = \frac{\int_{\tau}^{\delta} \left| F(s) - \bar{F}_m \right| ds}{\int_{\tau}^{\delta} F(s) ds} \tag{11}$$

The reusability ratio quantifies the average degree of overlap between the loading curves of two consecutive cyclic tests. As illustrated in Figure S4c, the blue shaded area indicates the deviation between the N-th and the first loading curves. Based on this deviation, the reusability ratio is defined as follows:

$$\text{Reusability ratio} = 1 - \left(\frac{\int_{0}^{l} \left| F_1(s) - F_N(s) \right| ds}{\int_{0}^{l} F_1(s) ds} \right)^{\frac{1}{N-1}} \tag{12}$$

Here, $F_1(s)$ and $F_N(s)$ represent the crushing forces during the first and N-th loading processes as functions of displacement, and l denotes the maximum displacement in the loading process.

When selecting energy-absorbing structures, higher strength is not always better; instead, we should choose the strength according to the specific protection target. The strength of an energy-absorbing structure must be lower than that of the protection target. For example, cork can be used to protect a hammer, but it is ineffective for protecting an egg. Moreover, the strength of the energy-absorbing structure should not be too low; otherwise, it cannot effectively fulfill its energy absorption function. As illustrated in Figure S4d, the red dashed line indicates the ultimate load capacity of the protected structure, denoted as F_{lim}. Intuitively, the ideal energy absorption curve should be a horizontal line slightly below F_{lim}, as represented by curve v in Figure S4d.

However, such an ideal energy absorption curve does not exist in reality; it can only be approached through rational structural design. Figure S4d presents four representative energy absorption curves. Curve i features no IPCF and exhibits no fluctuation in the plateau force. Curve ii shows significant plateau force fluctuation. Curve iii includes an IPCF but maintains a flat plateau force. Curve iv has no IPCF, but the plateau force increases progressively. Among them, curve i has the highest F_m/F_{max} value, indicating the greatest crushing force stability. It exhibits zero plateau force fluctuation and achieves the highest energy absorption, making it the closest attainable curve to the ideal curve v in reality.

The above analysis highlights the necessity of introducing the crushing force stability (F_m/F_{max}) and plateau force fluctuation as two essential indicators for evaluating energy absorption.

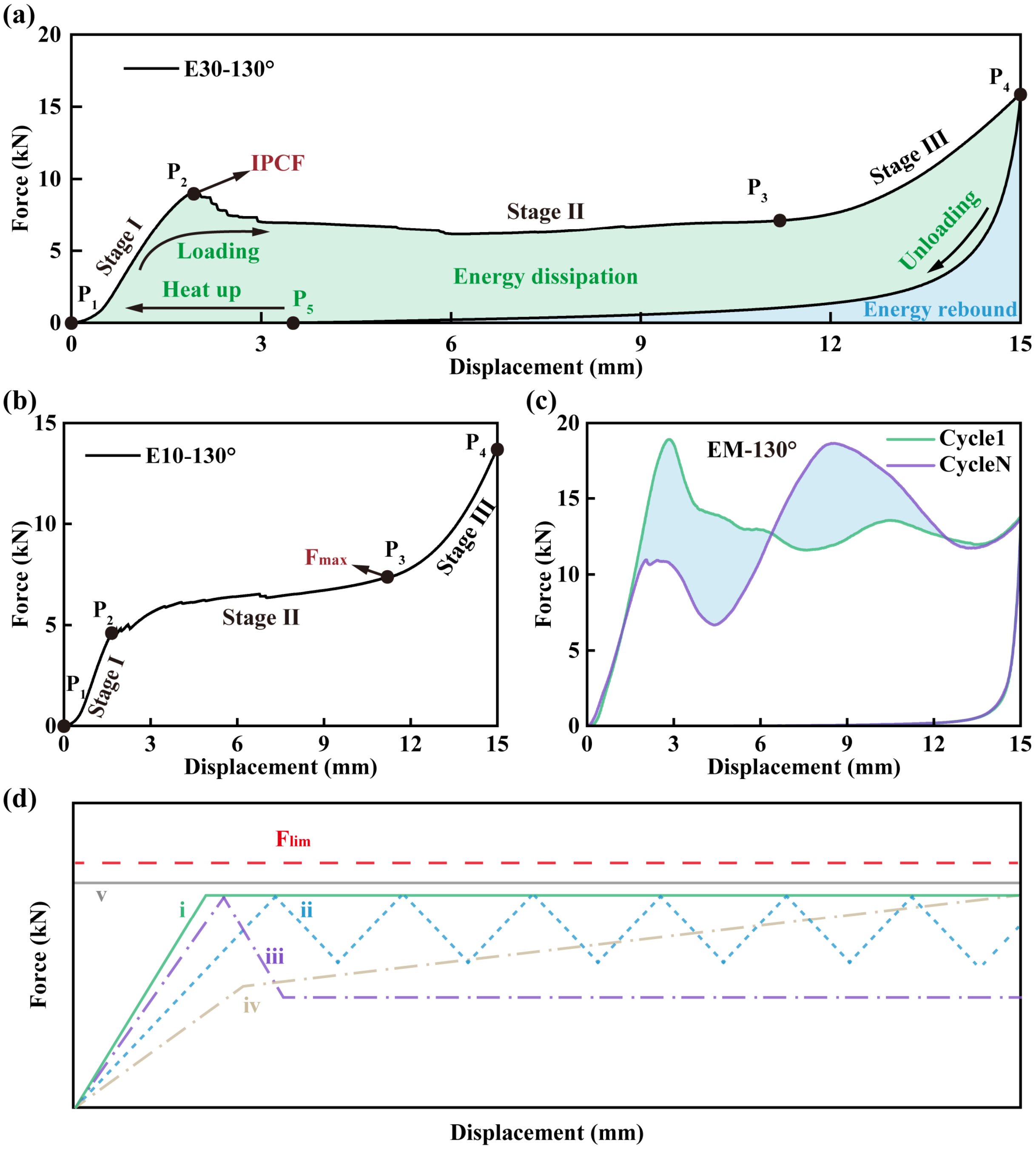

Figure S4. Representative force–displacement curves used to illustrate the energy absorption evaluation indicators. a) Force–displacement curve containing an IPCF, such as E30-130°, where F_{max} typically corresponds to the IPCF. b) Force–displacement curve without an IPCF, such as E10-130°, where F_{max} typically corresponds to the crushing force at the onset of the densification stage. c) Force–displacement curves of the first and N-th cycles for EM-130°. d) Four representative energy absorption curves and an ideal curve. Curve i features no IPCF and no fluctuation in the plateau stage. Curve ii exhibits significant fluctuation in the plateau stage. Curve iii contains an IPCF but exhibits no fluctuation in the plateau stage. Curve iv has no IPCF, but the force gradually increases during the plateau stage. Curve v represents the ideal energy absorption curve. The red dashed line indicates the maximum tolerable load of the protected

target.

S5: Force–Displacement Curves from Cyclic Tests at an LMPAO Thickness of 5 mm

At the LMPAO thickness t of 5 mm, the metal skeleton thickness d is 3 mm, as given by Equation (6). Figure S3 illustrates schematic diagrams of various structures with different geometric configurations, which were used for the cyclic tests, and **Figure S5** shows their corresponding force–displacement curves.

The force–displacement curves from the three-cycle tests exhibit high overlap as long as the width level of the metal skeleton is no greater than E40. Once the width level of the metal skeleton exceeds E40, the overlap of the force–displacement curves from the three-cycle tests drops sharply. The corresponding LMPAO exhibits poor reusability and can therefore be considered to have lost its reusable energy absorption function.

Within the range where the LMPAO retains its reusable energy absorption function, increasing the width of the metal skeleton enlarges the area enclosed by the loading–unloading curve. This indicates greater energy dissipation in a single cycle. Meanwhile, the area enclosed between the unloading curve and the displacement axis decreases. This means that less energy is released during rebound, which helps prevent secondary damage caused by structural rebound.

At width levels less than or equal to E20, the curves show no initial peak crushing force (IPCF), and the crushing force stability increases as the metal skeleton becomes wider. At width levels greater than E30, the curves exhibit an initial peak crushing force (IPCF). Moreover, as the width of the metal skeleton increases, the structural stiffness improves, which in turn elevates the buckling threshold. As a result, the IPCF of the curves rises, and the crushing force stability declines.

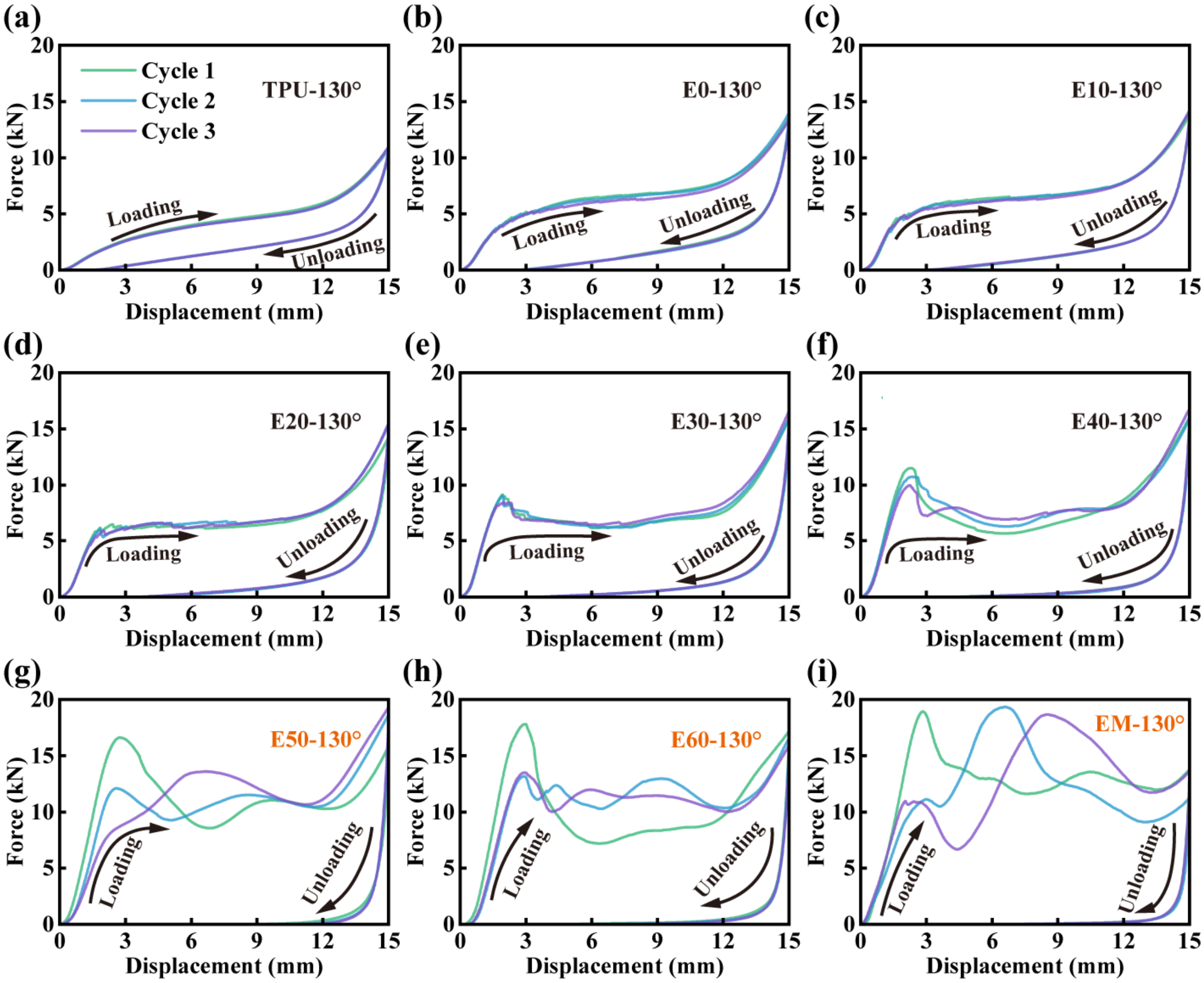

Figure S5. Force–displacement curves of LMPAO structures with different metal skeleton widths at a thickness of 5 mm. The orange-highlighted structures exhibit low overlap in their force–displacement curves, indicating a loss of reusable energy absorption capability.

S6: Force–Displacement Curves from Cyclic Tests at an LMPAO Thickness of 4.5 mm

At an LMPAO thickness t of 4.5 mm, the metal skeleton thickness d is calculated as 2.5 mm according to Equation (6). The schematics of the structures used for cyclic tests are illustrated in Figure S3, and their corresponding force–displacement curves are shown in **Figure S6.**

At this thickness, only E60-130° and EM-130° lose their reusable energy absorption capability. Compared with the LMPAO with a thickness of 5 mm, the range of reusable energy absorption is expanded. The influence of the metal skeleton width on energy dissipation, rebound energy, IPCF, and crushing force stability is consistent with that observed in the LMPAO with a thickness of 5 mm.

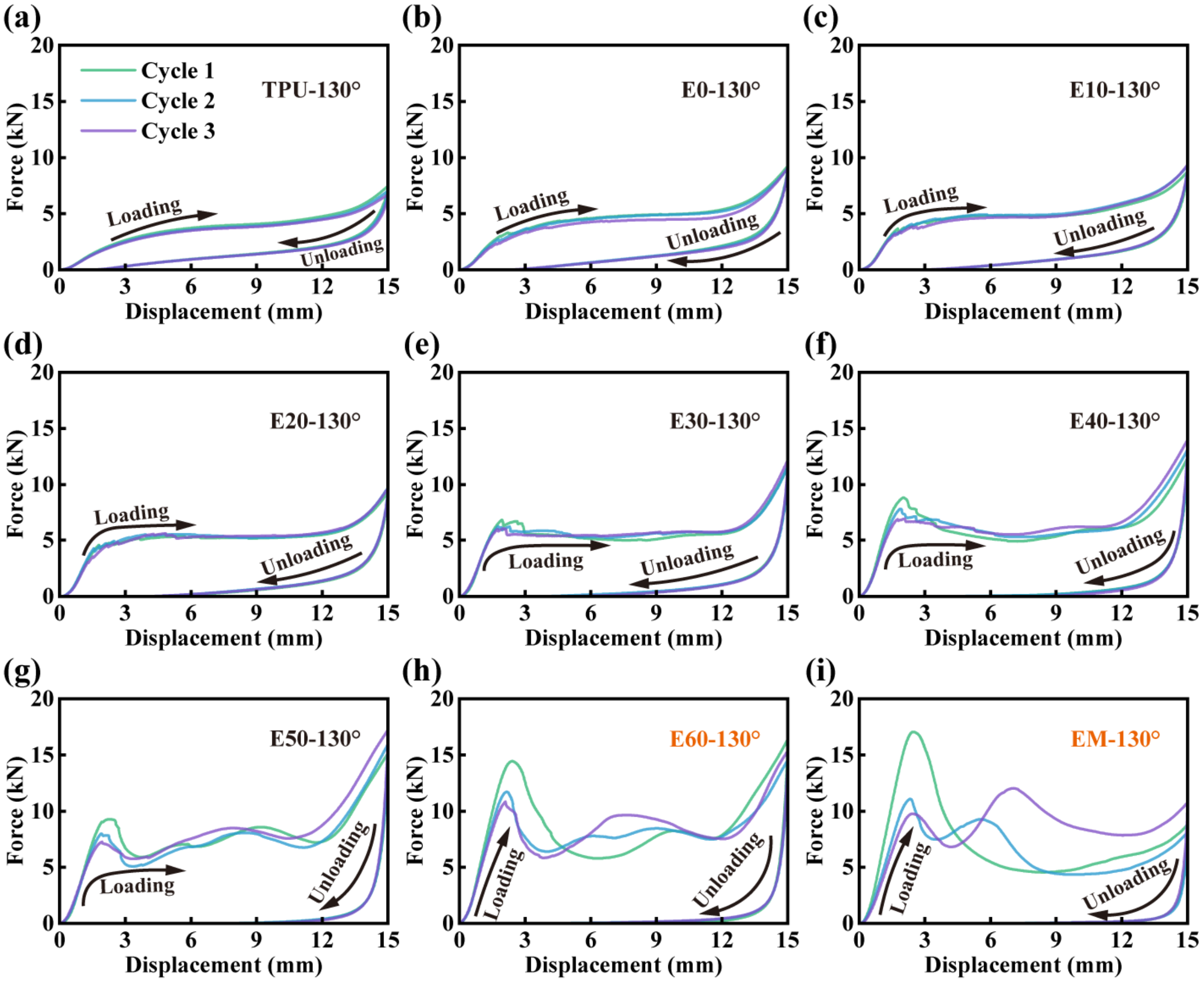

Figure S6. Force–displacement curves of LMPAO structures with different metal skeleton widths at a thickness of 4.5 mm. The orange-highlighted structures exhibit low overlap in their force–displacement curves, indicating a loss of reusable energy absorption capability.

S7: Force–Displacement Curves from Cyclic Tests at an LMPAO Thickness of 5.5 mm

At an LMPAO thickness t of 5.5 mm, the metal skeleton thickness d is calculated as 3.5 mm according to Equation (6). The schematics of the structures used for cyclic tests are illustrated in Figure S3, and their corresponding force–displacement curves are shown in **Figure S7**.

At this thickness, the structures E40-130°, E50-130°, E60-130°, and EM-130° lose their reusable energy absorption capability. Compared with the LMPAO with a thickness of 5 mm, the range of reusable energy absorption is reduced. The influence of the metal skeleton width on energy dissipation, rebound energy, IPCF, and crushing force stability is consistent with that observed in the LMPAO with a thickness of 5 mm.

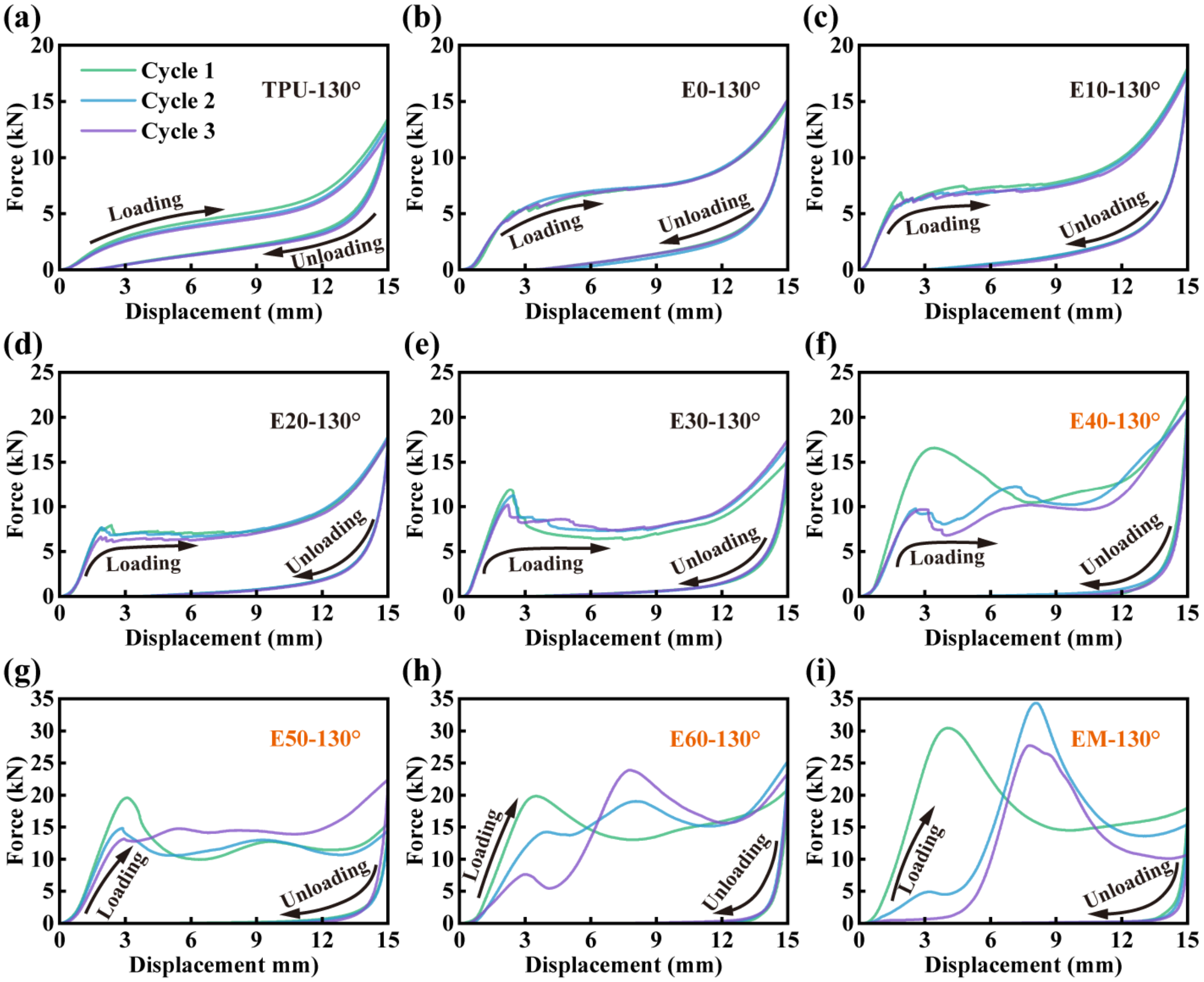

Figure S7. Force–displacement curves of LMPAO structures with different metal skeleton widths at a thickness of 5.5 mm. The orange-highlighted structures exhibit low overlap in their force–displacement curves, indicating a loss of reusable energy absorption capability.

S8: Force–Displacement Curves from Cyclic Tests of LMPAO with Different Dihedral Angles

We fixed the LMPAO thickness at 5 mm and set the metal skeleton width level to E20. Under this condition, cyclic tests were conducted on eight structures with dihedral angles ranging from 122° to 150°, as well as on a regular hexagonal structure (HEX). The corresponding force–displacement curves are shown in **Figure S8**.

At dihedral angles of 122° to 138°, the LMPAO exhibits the diamond deformation mode, and the corresponding experimental curves are shown in Figures S8a to S8e. The force–displacement curves contain no IPCF or only a negligible IPCF, and the crushing force stability is high. This represents an ideal mode for energy absorption. At a dihedral angle of 142°, the LMPAO still deforms such that the rhombic regions collapse inward while the trapezoidal regions expand outward, but the corresponding force–displacement curve shows a noticeable

IPCF (Figure S8f). Thus, this case can be regarded as a transitional deformation mode.

At dihedral angles of 146° and 150°, the deformation mode of the structure shifts to the expansion mode, and the corresponding force–displacement curves are shown in Figures S8g and S8h. The curves exhibit a distinct IPCF in the loading process, and the crushing force in the plateau stage is significantly lower than that of the diamond mode, which is unfavorable for energy absorption.

HEX (essentially the structure with a dihedral angle of 180°) corresponds to the force–displacement curve shown in Figure S8i. The loading process exhibits a pronounced IPCF, and the crushing force stability is low, making it unsuitable for serving as an energy-absorbing structure.

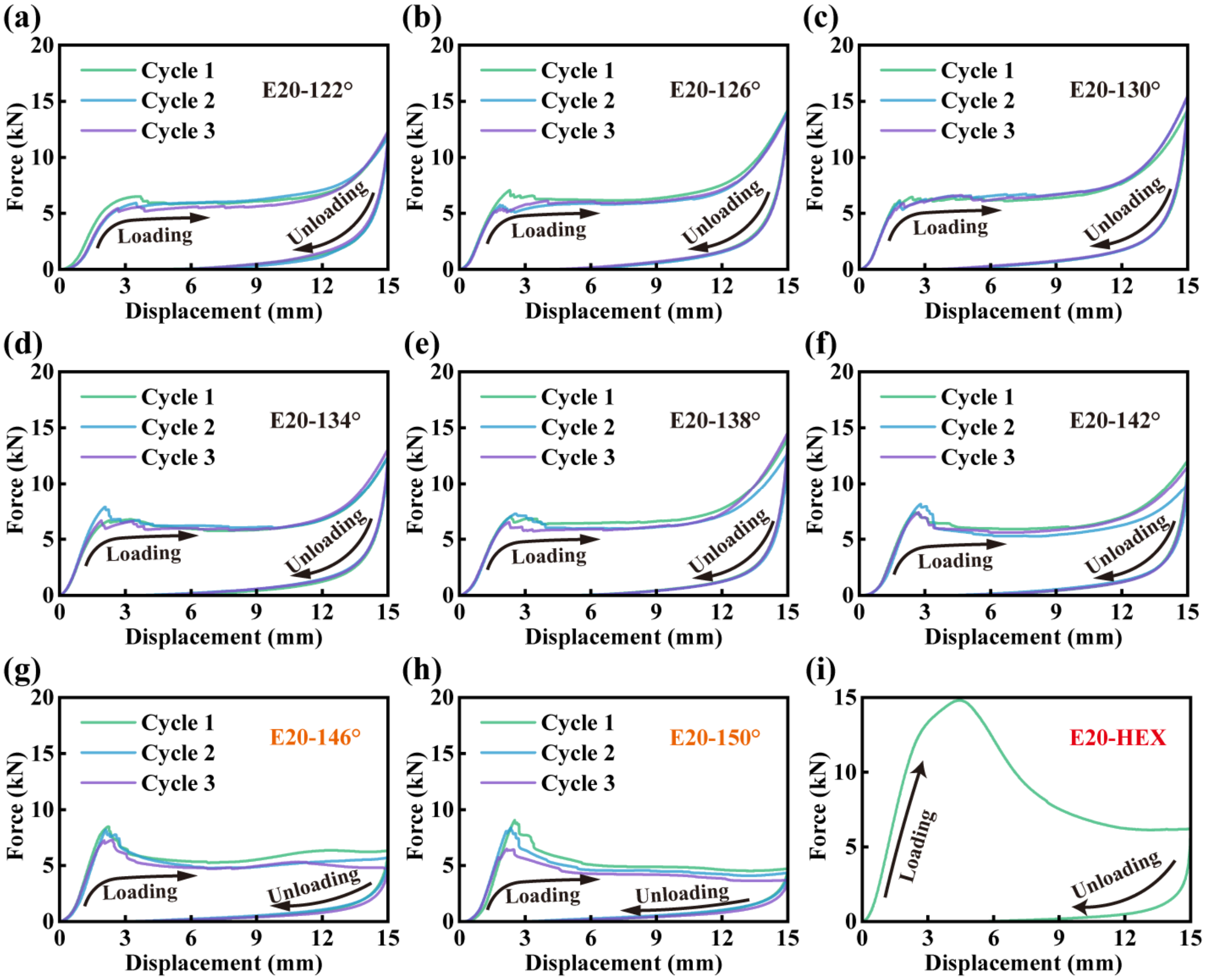

Figure S8. Force–displacement curves of LMPAO with different dihedral angles at a thickness of 5 mm. a)–e) Force–displacement curves of structures exhibiting the diamond deformation mode, showing no IPCF or only a negligible IPCF relative to the plateau stage, and high crushing force stability. f) Force–displacement curve of the structure exhibiting the transitional deformation mode. g)–h) Force–displacement curves of the structures exhibiting the expansion deformation mode, showing a pronounced IPCF and a significantly reduced load level during

the plateau stage. i) Force–displacement curve of the HEX structure.

S9: Mechanical responses of monolithic three-layer LMPAO before and after optimization

As shown in **Figure S9a**, the structure before optimization (E20-20-20-130°) exhibits a random deformation mode during the three cyclic compression processes. The corresponding force–displacement curves in Figure S9c show that this random deformation introduces randomness into the structural force response, significantly reducing the degree of curve overlap and resulting in insufficient reusability.

As shown in Figure S9b, during the three cyclic compression processes, the deformation of the optimized structure (E10-25-40-122°) is consistently guided by the creases and follows a stable diamond mode. The corresponding force–displacement curves in Figure S9d show that, under the diamond mode, the force response of the structure becomes more deterministic, resulting in a high degree of curve overlap and good reusability.

Figure S9e compares the reusability ratios before and after optimization. After optimization, the reusability ratio increases by 9.3 percentage points, demonstrating the superiority of the diamond deformation mode.

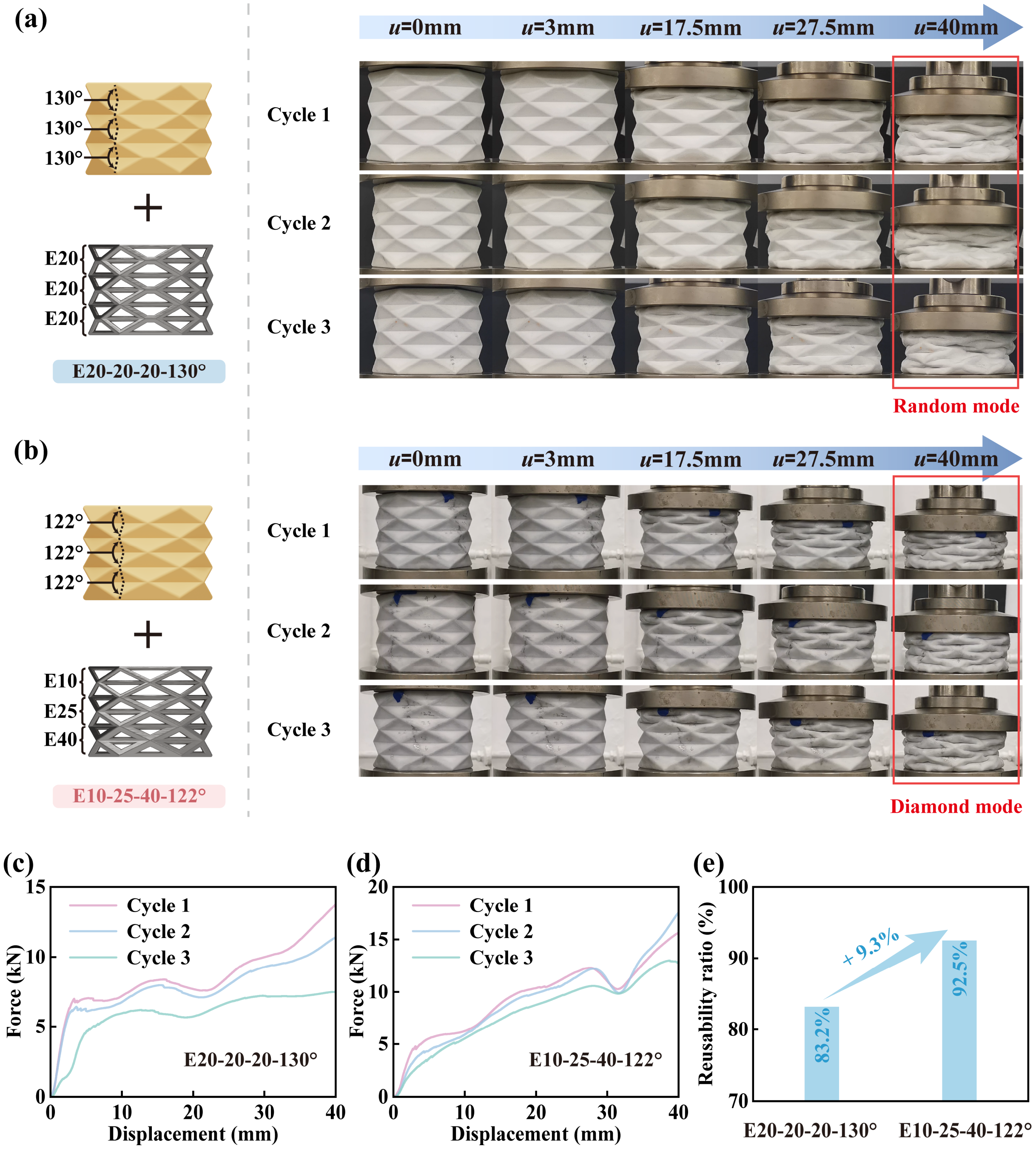

Figure S9. Mechanical responses of monolithic three-layer LMPAO before and after optimization. a) Experimental snapshots of E20-20-20-130° at key points during three cyclic compression processes. b) Experimental snapshots of E10-25-40-122° at key points during three cyclic compression processes. c) Force–displacement curves of E20-20-20-130° during three cyclic compression processes. d) Force–displacement curves of E10-25-40-122° during three cyclic compression processes. e) Reusability ratios of the structures before and after optimization.

S10: Geometric Dimensions of the Rigid Plates

To eliminate the deformation coupling between structural layers, we use grooved rigid plates to connect and constrain the unit cells. Two types of rigid plates are used. One type is placed at the top and bottom of the axially stacked structure, featuring grooves only on the side that contacts the LMPAO unit cells. Its front view is shown in **Figure S10a**, and the thickness is 3.6 mm. The other type is used for the axial connection of unit cells, featuring grooves on both sides. Its front view is shown in Figure S10b, and the thickness is 7.2 mm. Figure S10c shows the top view of the rigid plate. The outer boundary of the plate is a regular hexagon with a side length of 54 mm, and the inner boundary is a regular hexagon with a side length of 46 mm. The grooves are generated by a swept-cut operation, in which a semicircular profile with diameter D is swept along the path of a regular hexagon with a side length of 50 mm. The value of D depends on the LMPAO thickness t, ensuring that the bottom edge of the LMPAO unit cell can always be embedded into the groove.

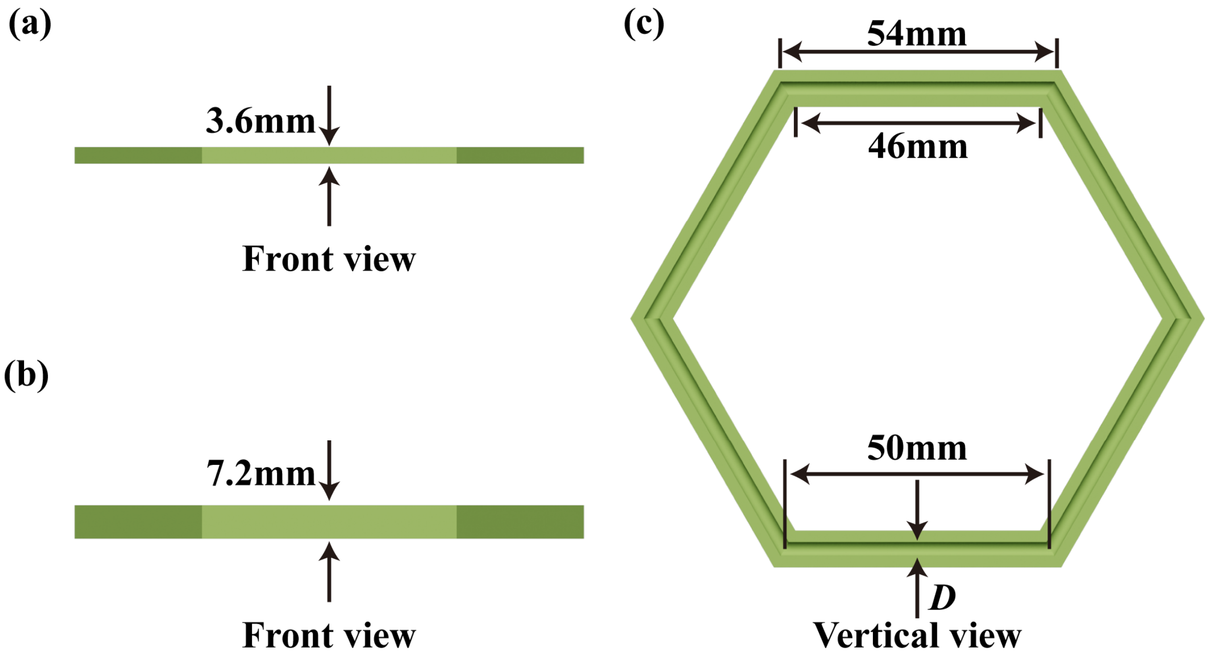

Figure S10. Geometric dimensions of the rigid plates. a) Front view of the top and bottom plates, with a thickness of 3.6 mm and grooves only on the side that constrains the LMPAO unit cells. b) Front view of the remaining plates, with a thickness of 7.2 mm and grooves on both sides. c) Top view of the rigid plate. The outer boundary of the plate is a regular hexagon with a side length of 54 mm, and the inner boundary is a regular hexagon with a side length of 46 mm. The grooves are generated by a swept-cut operation, in which a semicircular profile with diameter D is swept along the path of a regular hexagon with a side length of 50 mm.

S11: Quasi-static compression process of modular multilayer LMPAO

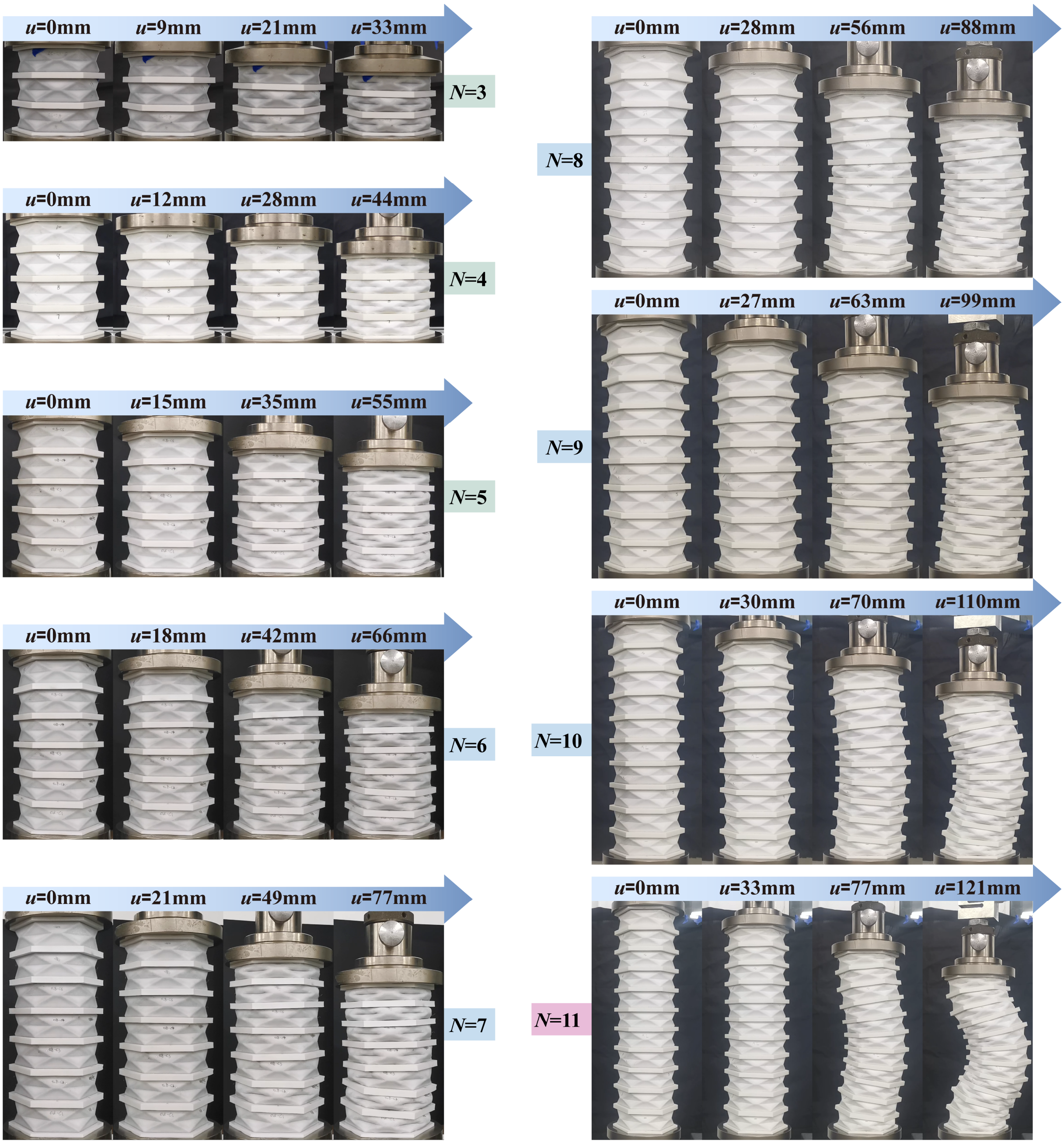

Figure S11. Experimental snapshots at key points during the quasi-static compression process of modular multilayer LMPAO

S12: Force–displacement curves of modular multilayer LMPAO during compression

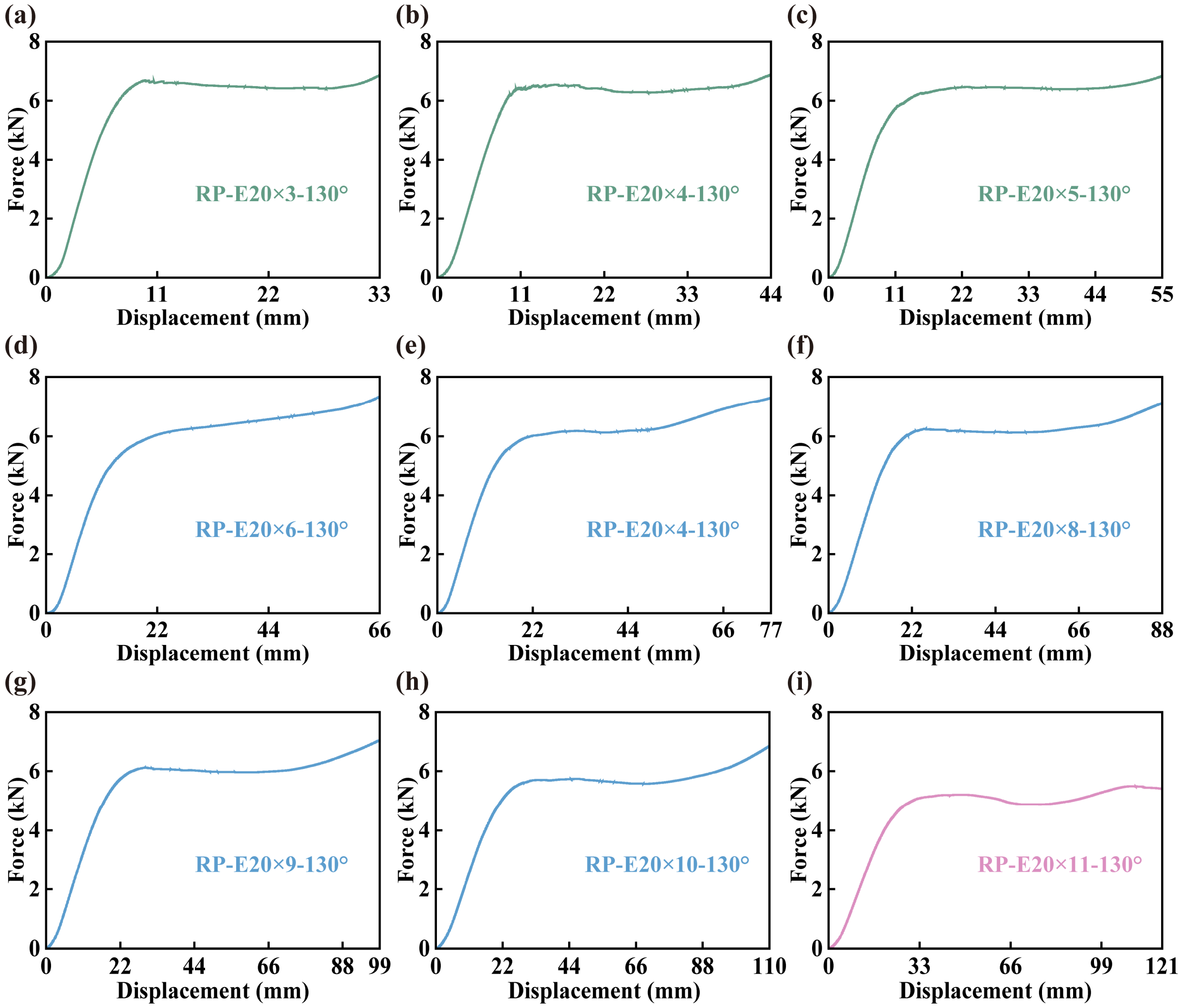

Figure S12. Force–displacement curves of modular multilayer LMPAO during compression.

S13: Definitions of the reference area *A* and volume *V* used for normalization

In *EA*/*A* and *EA*/*V*, *A* denotes the projected area of the structure, and *V* denotes the structural volume. Taking the five-layer modular LMPAO shown in **Figure S13** as an example, *A* is defined as the area of the regular hexagonal rigid plate with a side length of 54 mm, and *V* is calculated as the structural height *H* multiplied by the projected area *A*.

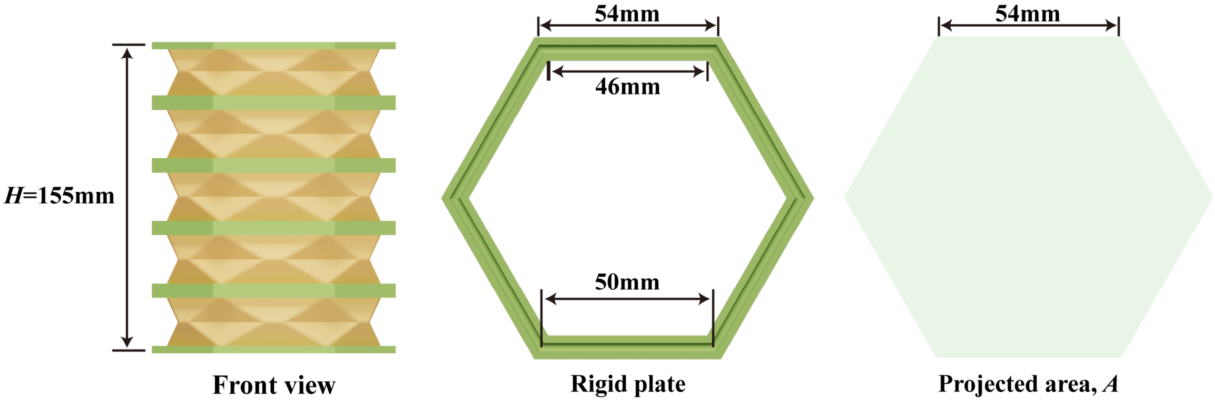

Figure S13. Schematic illustration of the definitions of the projected area *A* and structural height *H* in the five-layer modular LMPAO.

S14: Tensile Samples and Engineering Stress–Strain Curves of TPU-95A and InSnBi Alloy

TPU is the abbreviation for thermoplastic polyurethane elastomer rubber, which features a wide hyperelastic range. In this work, TPU filament with a Shore hardness of 95A is used to 3D print the TPU shell of the LMPAO.

InSnBi is a type of low-melting-point alloy (LMPA), whose melting point is determined by the composition of its constituent metals. In this work, an InSnBi alloy with a melting point of 47 °C is used to construct the metal skeleton of the LMPAO. Although the energy absorption capacity of the InSnBi alloy is lower than that of engineering metals, it is significantly higher than that of typical flexible materials. The tensile samples and engineering stress–strain curves of TPU-95A and the InSnBi alloy are shown in **Figure S14**.

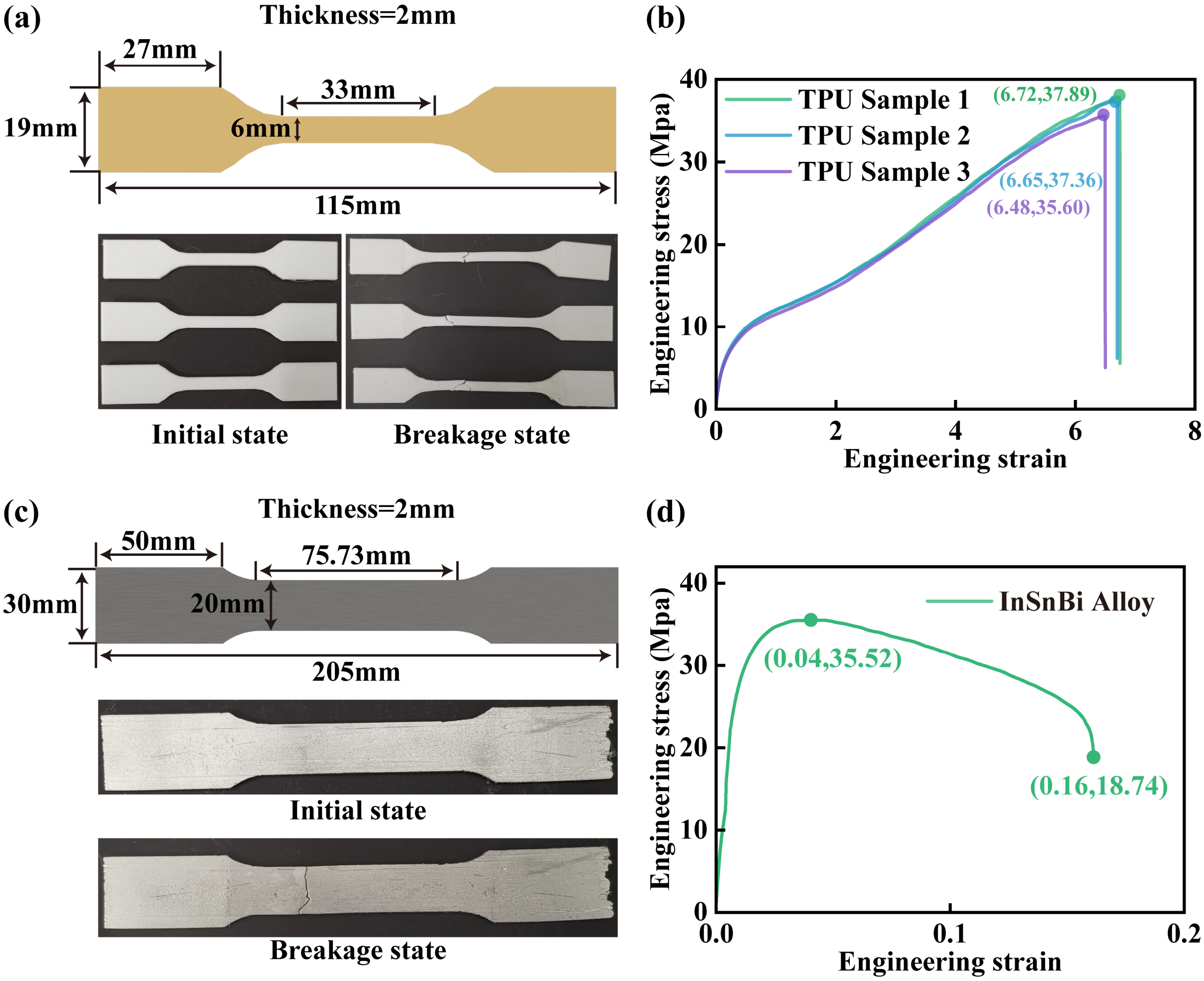

Figure S14. Tensile samples and engineering stress–strain curves of TPU-95A and the InSnBi alloy. a) Dimensions of the dog-bone-shaped TPU-95A sample and its initial and breakage states. b) Engineering stress–strain curve of TPU-95A. c) Dimensions of the dog-bone-shaped InSnBi alloy sample and its initial and breakage states. d) Engineering stress–strain curve of the InSnBi alloy.

S15: Ashby plot using SEA to characterize energy absorption capacity

As shown in **Figure S15,** the LMPAO exhibits good crushing force stability and reusability, but its SEA is slightly lower than that of some metamaterials. This indicates that the introduction of the metal skeleton indeed brings a certain mass penalty, making the lightweight advantage of the LMPAO less pronounced. However, among the existing comparable structures, no reusable energy-absorbing metamaterial outperforms the LMPAO across all three performance dimensions. Therefore, the comparison based on the SEA indicator does not overturn the core conclusion of this study. It should be noted that most studies included in Figure 7 do not report the sample mass, resulting in a limited number of data points available for SEA calculation.

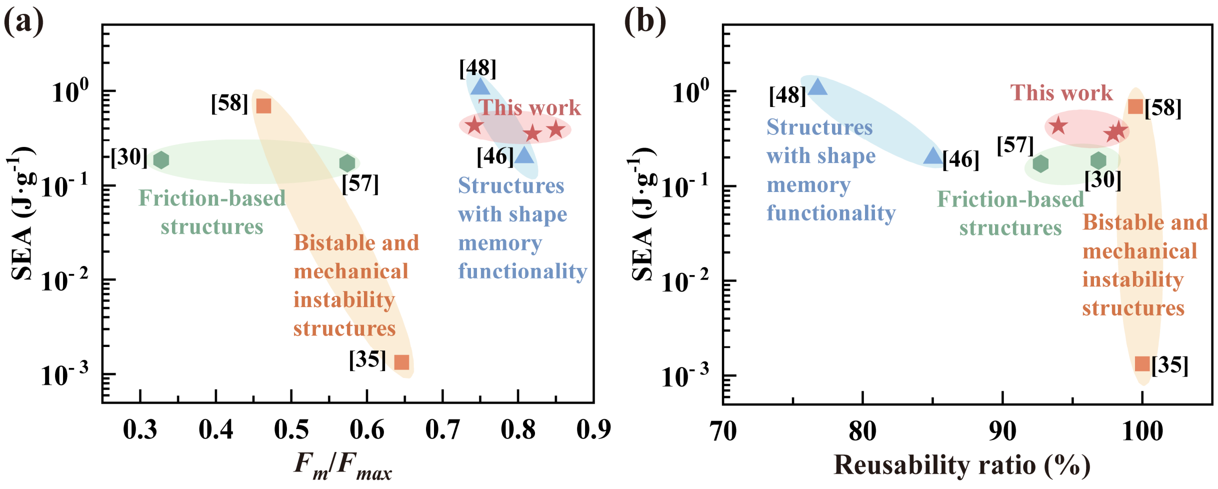

Figure S15. a) Comparison in terms of SEA and crushing force stability. b) Comparison in terms of SEA and reusability.

S16: Design of Dual-Plateau Modular LMPAO Metamaterials

In practical applications, structures may need multiple plateau segments to satisfy the energy absorption requirements under complex loading conditions. Eliminating deformation coupling between structural layers allows each layer to function as an independent deformation module, thereby enabling modular design of multilayer LMPAO metamaterials. Meanwhile, adjusting the thickness of the LMPAO unit cell, as shown in Section 2.2, allows tailoring the force plateau height of each module. Therefore, this assembly strategy enables the tailoring of energy absorption curves with multiple plateau characteristics.

Figure S16 presents the mechanical response of the LMPAO metamaterial with dual-plateau characteristics. The width level of the metal skeletons is E20 for both the upper and lower unit cells, and the dihedral angle is 130° for both. The thickness of the upper unit cell is 4.5 mm, and that of the lower unit cell is 5.5 mm. As compression progresses, the upper unit cell collapses first, resulting in a relatively low plateau stage in the force–displacement curve.

After the upper unit cell enters the densification stage, the lower unit cell begins to collapse, leading to a relatively high plateau stage.

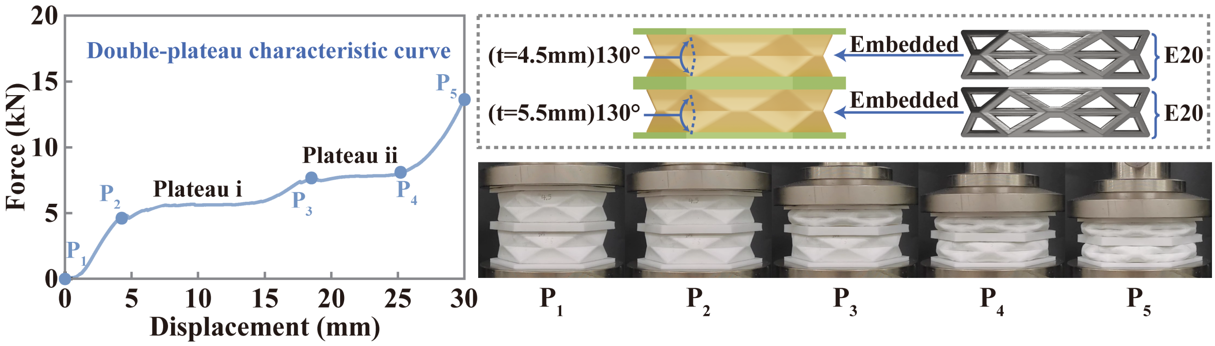

Figure S16. LMPAO metamaterial with dual-plateau characteristics.

S17: Recovery mechanisms of existing reusable energy-absorbing metamaterials

Among the reusable energy-absorbing metamaterials included in the Ashby plots in Figure 7, only some friction-based structures ([27], [56], [57]) and one structure based on mechanical instability ([59]) can achieve passive elastic recovery, whereas the other metamaterials rely on active recovery mechanisms.

For structures based on SMA and SMP, the constituent materials themselves exhibit a shape memory effect, and recovery is usually thermally triggered. For metamaterials based on the bistable mechanism, the structures possess two stable configurations. After energy absorption, they usually remain in the second stable state and cannot achieve passive recovery. Recovery to the first stable state can only be achieved by applying reverse loading to provide the energy required for the structure to cross the energy barrier. For structures based on the friction mechanism, the recovery behavior is more complex. After unloading, if the frictional resistance exceeds the elastic restoring force, the structure will be locked and require reverse loading for recovery; if the frictional resistance is lower than the elastic restoring force, the structure can overcome the frictional resistance and recover passively. Structures based on mechanical instability can form hysteresis loops by axially stacking a large number of buckling units, thereby achieving passive elastic recovery.

S18: Service life of the LMPAO metamaterial

To evaluate the service life of the LMPAO, we increased the number of cycles and continued the test until local tearing occurred in the TPU shell. As shown in **Figure S17a**, this local tear occurs after the 37th cycle. Because the LMPA is liquid during thermal recovery, the torn TPU shell can no longer effectively contain the liquid metal. Therefore, shell tearing can

be regarded as the endpoint of the cyclic service life of the LMPAO.

Figure S17b shows the force–displacement curves of all 37 cycles, which exhibit a high degree of overlap. Figure S17c further compares the curves of the 1st, 19th, and 37th cycles, showing a slight decrease in the plateau force with increasing cycle number. Figure S17d presents the energy dissipation in each cycle. In the 37th cycle, the energy dissipation remains at 85.5% of that in the 1st cycle. These results indicate that the LMPAO maintains good reusability before tearing occurs in the TPU shell. However, it has a limited cyclic service life and cannot be reused indefinitely.

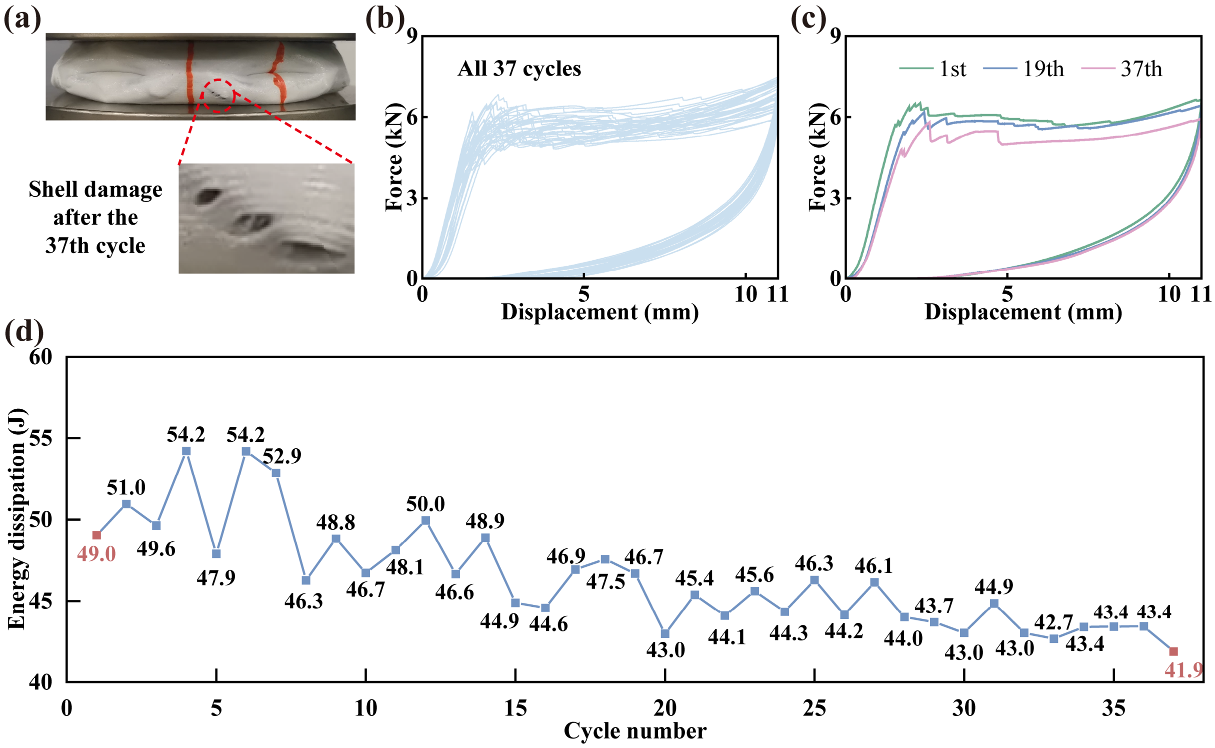

Figure S17. Mechanical responses of the LMPAO during its service life. a) Appearance of the LMPAO after shell tearing. b) Force–displacement curves from 37 cyclic tests. c) Force–displacement curves of the 1st, 19th, and 37th cycles. d) Energy dissipation in each cycle.
